# The Speech Network in Childhood Stuttering: Differences in Functional Connectivity of the Planning and Motor Loops

**DOI:** 10.1162/NOL.a.26

**Published:** 2026-01-13

**Authors:** Fiona Höbler, Yanni Liu, Adriene M. Beltz, Hannah C. Becker, Mike Angstadt, Frank H. Guenther, Soo-Eun Chang

**Affiliations:** Department of Psychiatry, University of Michigan, Ann Arbor, MI, USA; School of Communication Sciences and Disorders, Western University, London, ON, Canada; Department of Speech, Language, and Hearing Sciences, Boston University, Boston, MA, USA; Department of Communication Disorders, Ewha Womans University, Seoul, South Korea

**Keywords:** basal ganglia thalamocortical circuit, developmental stuttering, network analysis, resting-state functional connectivity, speech motor network

## Abstract

Developmental stuttering is a complex neurodevelopmental condition associated with structural and functional anomalies in the basal ganglia-thalamo-cortical (BGTC) circuits that support speech planning and execution. In this study, we examined hypothesized impairments in the planning and motor circuits of the speech network in children who stutter (CWS), compared to children who do not stutter (CNS), using person-specific functional connectivity maps derived from resting-state functional magnetic resonance imaging (rsfMRI) data. RsfMRI data were acquired from 73 CWS and 74 CNS, aged 3 to 10 years. Twelve regions of interest within the speech motor networks were extracted. Functional connectivity was assessed using confirmatory subgrouping group iterative multiple model estimation (CS-GIMME), which estimates group-, subgroup-, and individual-level connections. Subgroup-level functional connectivity patterns revealed altered connections among CWS in both planning and motor loops, including reduced within-network connectivity, compared to CNS. CWS showed connectivity between the left posterior inferior frontal sulcus and left ventral lateral thalamus that was not observed in CNS. Furthermore, centrality of the left ventral lateral thalamus and right ventral premotor cortex were increased in CWS relative to CNS. Significant differences between CWS and CNS in within-network connectivity highlight early developmental alterations that affect the BGTC circuitry, pointing toward inefficiencies in the neural network that supports the programming, planning and timing of speech motor sequences.

## INTRODUCTION

Developmental stuttering emerges in early childhood during a heightened period of speech motor skill acquisition (Meier & Guenther, 2023; Ostry et al., 1984; Smith, 2006), estimated to affect 5%–7% of children and to persist in approximately 1% of the population (Yairi & Ambrose, 1999, 2013). As a complex neurodevelopmental condition (Ludlow, 2000; Smith & Weber, 2017), developmental stuttering has been characterized by systems-level impairments evidenced in both behavioral and neuroimaging research (Cai, Tourville, et al., 2014; Chang & Guenther, 2020). On a behavioral level, individuals who stutter have shown difficulties in auditory-motor integration needed for the timely adjustment of articulators during motor planning and control of speech production (Cai, Beal, et al., 2014; Cai et al., 2012; Daliri et al., 2018). These impairments have been variously interpreted as indicators of deficiencies in the sensorimotor control system for online speech sequencing among persons who stutter (Cai, Beal, et al., 2014), as instabilities in the internal models used for feedback and feedforward control systems (Max et al., 2004), or as an inefficient overreliance on sensory feedback mechanisms (Max et al., 2004). The neuroimaging evidence has pointed toward structural and functional anomalies among children and adults who stutter in regions and connections that form the basal ganglia-thalamo-cortical (BGTC) circuit, which supports the planning and execution of speech motor sequences (Bohland et al., 2019; Guenther, 2016). Overall, this evidence has led to the hypothesis that systems-level impairments in the BGTC circuit implicate the ability of individuals who stutter to produce the necessary timing cues for the initiation of speech motor segments, manifesting as repetitions, prolongations, or blocks that interrupt fluent speech sequencing (Alm, 2004; Chang & Guenther, 2020; Civier et al., 2013; Guenther, 2016).

### Basal Ganglia-Thalamo-Cortical Circuits and Developmental Stuttering

Despite the complexity of the stuttering experience, and growing evidence in support of systems-level impairments associated with the condition, the majority of research into the neural basis of developmental stuttering has focused on localizing neurophysiological differences between persons who do and do not stutter through region-specific analyses (Belyk et al., 2015, 2017; Brown et al., 2005; Etchell et al., 2018). These studies have revealed differences in structural brain development between children who do (CWS) and do not stutter (CNS) across several cortical regions in the left hemisphere that are critical to speech motor production. Reduced gray matter volume (GMV) was identified in the left inferior frontal gyrus (IFG; Beal et al., 2013; Chang et al., 2008; Chow et al., 2023), precentral gyrus (Chang et al., 2008; Garnett et al., 2018) and adjacent regions, including the premotor (PMC; Garnett et al., 2018), supplementary motor (SMA; Chang et al., 2008), and primary motor areas (M1; Garnett et al., 2018). The inferior frontal sulcus (IFS) is involved in speech and language, as well as working memory processes, with speech and language functions showing stronger left lateralization (Holland et al., 2007; Ruland et al., 2022). The SMA projects to M1 and PMC for the programming and control of movements (Alexander et al., 1986). Together with the subcortical structures of the basal ganglia and thalamus, these cortical regions form functional circuits that enable the timely selection of correct movement responses within the current behavioral context (Guenther, 2016; Mink, 1996). The striatal structures of the caudate (Foundas et al., 2013; Miller et al., 2023) and putamen (Beal et al., 2013; Chow et al., 2023), along with the thalamus (Chow et al., 2023) have also exhibited reduced GMV in CWS when compared to CNS, suggesting that these regional differences may contribute to inefficiencies in the programming, planning, and selection of speech movements among CWS, processes that are supported by the cortico-basal ganglia-thalamo-cortical network (Chang & Guenther, 2020; Chow et al., 2023; Foundas et al., 2013). As a result, individuals who stutter are thought to rely more heavily on sensory feedback processing in the auditory cortex (Max et al., 2004), with greater GMV in the left superior temporal gyrus (STG) observed in CWS compared to CNS (Beal et al., 2015).

Further support for this hypothesis has been provided in a recent longitudinal investigation of brain development in CWS, showing that reduced growth rates of GMV in the left inferior frontal and ventral premotor regions, as well as reduced growth of white matter (WM) volume interconnecting these areas, were associated with stuttering persistence (Chow et al., 2023). Accordingly, adults with persistent developmental stuttering also exhibit GMV differences in the left IFG (Beal et al., 2007, 2015; Kell et al., 2009; Lu et al., 2012) and precentral gyrus (Beal et al., 2007), along with the bilateral STG (Beal et al., 2007) and striatal structures of the left putamen (Lu et al., 2010) and caudate nucleus (Sowman et al., 2017). Although the direction of GMV differences in the inferior frontal regions has not been consistent among adults who stutter (AWS; Etchell et al., 2018), these neuroanatomical distinctions associated with stuttering have been further supported by evidence of reduced WM structural connectivity, as measured by fractional anisotropy (FA), between the IFG, motor cortices, and auditory cortex in the STG of the left hemisphere (Chang et al., 2011; Cykowski et al., 2010; Lu et al., 2010; Sommer et al., 2002). Conversely, WM connectivity between homologous regions in the right hemisphere was found to be increased among AWS (Beal et al., 2007; Chang et al., 2011; Jäncke et al., 2004; Lu et al., 2010), with structural connectivity of hyperactive frontal regions, including the right inferior frontal and precentral gyri, along with the anterior thalamic radiation, positively associated with stuttering severity among adults with persistent stuttering (Neef et al., 2018). The summation of reported neuroanatomical differences seen across these cortical and subcortical regions, as well as in the connectivity between them, points toward significant systems-level differences among both children and adults who persist to stutter in the BGTC circuit that supports the speech motor network.

### Neural Network Models of Speech Motor Skill Acquisition and Production

**Figure 1.**
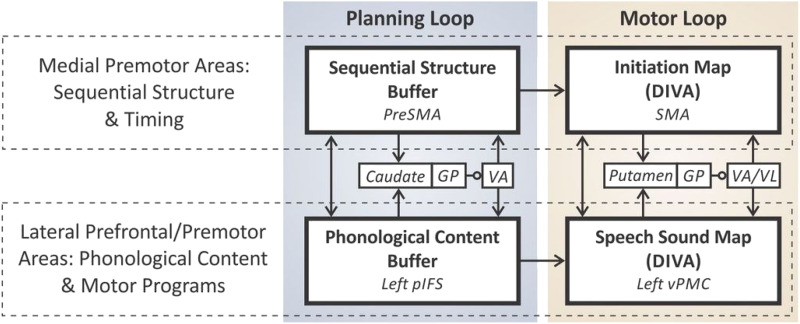
Schematic of the gradient order directions into velocities of articulators (GODIVA) model of speech sequence production and its neural correlates (adapted from Guenther, 2016, chapter 8). *Abbreviations*: PreSMA = pre-supplementary motor area; GP = globus pallidus; VA = ventral anterior nucleus of the thalamus; pIFS = posterior inferior frontal sulcus; SMA = supplementary motor area; VL = ventral lateral nucleus of the thalamus; vPMC, ventral premotor cortex.

Brain regions within the BGTC circuit that are relevant to speech motor control have been modeled as part of the directions into velocities of articulators (DIVA) neurocomputational model of speech motor skill acquisition and production. The DIVA model includes nodes from the cortical and subcortical regions that form interactive feedback and feedforward subsystems to support the neural control of speech production (Guenther, 2016). An important extension of this model was provided with the gradient order DIVA (GODIVA) model, which brings together the neural networks for language and speech motor control, with two parallel systems responsible for the planning and initiation of speech sounds (Bohland et al., 2010). As illustrated in Figure 1, the *planning loop* is formed by a sequential structure buffer in the preSMA and a phonological content buffer in the left posterior IFS (pIFS), which interact via direct connections as well as via a BGTC loop involving the caudate, globus pallidus, and ventral anterior thalamus. This speech planning process relies on phonological working memory to buffer upcoming speech sounds (Bohland et al., 2010; Guenther, 2016), while the *motor loop* initiates and programs the speech sequence by connecting the initiation map in the SMA to the speech sound map in the left ventral premotor cortex (vPMC) via projections to the putamen, globus pallidus, and thalamus (Bohland et al., 2010; Guenther, 2016). Additionally, cross-circuit connections between the medial premotor areas of preSMA and SMA activate and deactivate the initiation of motor programs in the intended order and with appropriate stress, thereby controlling the tempo of sequential utterances (Meier & Guenther, 2023). Further cortical connections between the lateral prefrontal and premotor areas of the left pIFS and vPMC in each circuit choose the next motor program to be executed based on the highest-activity node in the pIFS’s phonological buffer (Meier & Guenther, 2023).

During speech motor learning, the number of activated nodes in cortical regions of the pIFS, preSMA, and vPMC decrease, while subcortical connectivity involving the cerebellum and basal ganglia increases in order to support the more automated execution of speech motor sequences (Masapollo et al., 2021; Meier & Guenther, 2023; Segawa et al., 2015). With most of the DIVA and GODIVA nodes implicated in developmental stuttering, moments of stuttering are hypothesized to arise from specific disruptions to the functioning of the basal ganglia proper (Alm, 2004, 2021; Civier et al., 2013), or in corticostriatal WM connectivity (Civier et al., 2013), thereby impacting basal ganglia signaling and the automatized processing of speech (Alm, 2004, 2021; Chang & Guenther, 2020). Alternatively, functional differences at the cortical level may lead to inefficiencies and an overreliance on the less effective feedback control system that monitors sensory feedback for error-based processing (Civier et al., 2010), thereby also preventing more efficient and automated feedforward execution of speech (Chang & Guenther, 2020; Max et al., 2004). In support of hypothesized disruptions in corticostriatal connectivity, a recent meta-analysis of functional connectivity studies with children and adults who do and do not stutter (Matsuhashi et al., 2023) found reduced functional connectivity among both CWS and AWS, most prominently between the putamen and SMA. Within the DIVA circuitry, the putamen is hypothesized to monitor sensorimotor signaling for the appropriate context that cues the production of the next speech sound (Guenther, 2016). The pathway between the putamen and SMA provides input to the motor loop of the GODIVA neural network (Bohland et al., 2010) and points toward potential disruptions in the circuitry supporting the timely initiation of speech sound sequencing during speech production.

To date, network-level analyses among individuals who stutter have focused on neurocomputational simulations of stuttering (Civier et al., 2013), structural and connectomic analyses among adults with persistent stuttering (Cai, Tourville, et al., 2014; Gracco et al., 2022; Neef et al., 2018; Sitek et al., 2016; Theys et al., 2024), and the whole-brain analysis of large-scale neural network connectivity in CWS (Chang et al., 2018). These previous network-based analyses found reduced connectivity of nonmotor regions with the cerebellum and between the hemispheres among AWS (Gracco et al., 2022), as well as reduced connectivity within the default mode (Chang et al., 2018; Gracco et al., 2022), and internetwork connectivity among the somatomotor and the dorsal and ventral attention networks in individuals who stutter (Chang et al., 2018). Developmental stuttering has also been associated with structural differences in regions that support auditory integration within the speech motor network (Watkins, 2011), with reports of reduced connectivity within the networks of CWS supporting the timing of self-paced movement (Chang & Zhu, 2013) and hyperconnectivity between the supramarginal gyrus and prefrontal cortex in adults with persistent stuttering (Kell et al., 2018). A recent study of neural network mapping found overlap of network-level impairments associated with the left putamen, claustrum and striatal connectivity between adults experiencing stuttering since childhood and those having acquired stuttering following stroke (Theys et al., 2024). Additionally, increased connectivity between frontal and subcortical regions that participate in several neural networks in the right hemisphere has been associated with more frequent stuttering in adults (Neef et al., 2018), while greater functional connectivity between the left cerebellum and left prefrontal cortex was associated with less frequent stuttering among AWS (Sitek et al., 2016). These findings suggest that reduced connectivity within the left lateralized speech motor network of persons who stutter may be associated with the persistence of stuttering, whereas impaired connectivity within and between broader functional networks points toward neurodevelopmental anomalies that go beyond speech motor production, implicating the neural mechanisms and resources that support attentional control (Chang et al., 2018; Donaher & Richels, 2012; Tichenor et al., 2021). Critically, how the regions that are essential to speech motor planning and execution form this functional network during childhood is still unknown.

### Functional Connectivity Mapping Using GIMME

Although their findings highlight the broader implications of developmental stuttering to whole-brain network connectivity, previous efforts have been limited by their reliance on relatively small sample sizes (Gracco et al., 2022) and on methods that apply averaged group data without taking individual heterogeneity into account (Chang et al., 2018; Matsuhashi et al., 2023; Neef et al., 2018). The protracted process of neurodevelopment, and neural network specialization in particular, is strongly influenced by genetic variation as well as highly individualized experiences and environmental effects, which lead to inherently heterogeneous outcomes (Molenaar, 2004). As is the case for most populations of clinical interest, developmental stuttering is highly variable in its overt presentation, developmental course, treatment responsiveness, and its neural characterization (Baxter et al., 2016; Constantino et al., 2016, 2020; Etchell et al., 2018; Ludlow & Loucks, 2003; SheikhBahaei et al., 2023; Smith & Weber, 2017). Fortunately, a growing number of person-specific network-based analyses can be used to accurately model the inherent heterogeneity and extensive variability of psychological processes across individuals, leading to a more advanced understanding of complex neurodevelopmental and psychiatric conditions (Gates et al., 2014; Molenaar, 2004).

Group iterative multiple model estimation (GIMME), one person-specific approach, has proven to be both valid and reliable as a data-driven method of automated network modeling that can be applied to resting-state neuroimaging data to identify sparse and directed functional connectivity maps (Beltz & Gates, 2017; Gates & Molenaar, 2012). It achieves this by fitting unified structural equation models that contain both first-order lagged and time-locked contemporaneous connections (Gates et al., 2010). The derived person-specific networks contain group-level, potentially subgroup-level, and individual-level connections to reflect commonalities in network activity across groups, individuals, or connections that may be specific to an individual (Beltz & Gates, 2017; Gates & Molenaar, 2012; Gates et al., 2014). Thereby, GIMME can estimate individual-level, as well as group- and subgroup-level sparse connectivity patterns that account for heterogeneity among individual participants, going beyond the previous limitations of alternative methods that rely on group averaging. This approach has performed well in large-scale simulations (Gates & Molenaar, 2012; Henry et al., 2019; Lane et al., 2019) and has led to novel insights in psychiatric research, including neural network development and behavioral outcomes in adolescents at risk for substance use disorders (Mattoni et al., 2023), treatment-induced changes in task-based connectivity patterns in persons with obsessive-compulsive disorder (Becker et al., 2024), as well as identifying heterogeneous substrates of depression among affected adults (Price et al., 2017).

In the current study, we used the confirmatory subgrouping extension of GIMME, namely, CS-GIMME (Henry et al., 2019). By applying CS-GIMME to resting-state functional magnetic resonance imaging (rsfMRI) data, we sought to estimate subgroup-specific connections within the speech motor network for our a priori groups (CWS and CNS). This involved the identification of connections between nodes (or *edges*) that are present for the majority of individuals within the CWS and CNS groups. We also tested for hypothesized impairments in the speech network of CWS, reflecting individual-specific heterogeneity in connectivity. Using each participant’s network estimated by CS-GIMME, we determined within-network density (i.e., the total number of connections between regions of interest [ROIs] in the same network relative to the total number of connections estimated for each participant), and node centrality (i.e., the number of connections for each ROI relative to the total number of connections estimated for that participant), reflecting the contribution of each ROI to network function.

We hypothesized that functional connectivity patterns detected with CS-GIMME would show distinct subgroup-level network differences between CWS and CNS within the planning and motor loops of the GODIVA (Bohland et al., 2010), as well as in the broader network of speech production *feedback* and *feedforward circuit* structures of the DIVA model (Guenther, 2016; Guenther & Vladusich, 2012; Tourville & Guenther, 2011). Specifically, we hypothesized that within-network density would be significantly decreased in the planning and motor circuits of CWS, when compared to that of CNS. If developmental stuttering were to arise primarily from inefficiencies or disruptions in the GODIVA planning loop, then within-network connectivity involving the preSMA, left pIFS, and left caudate nucleus may be significantly reduced in CWS, or most significantly among a subset of CWS who persisted to stutter (CWSp). In the GODIVA motor loop, the SMA’s initiation map relies on optimized connectivity with the putamen (PUT) and the ventral anterior nucleus of the thalamus for the automated readout of learned movement sequence or phonemic chunks, which form optimized motor programs following successful speech motor acquisition (Meier & Guenther, 2023). Notably, impairments within the basal ganglia are thought to be a core deficit in developmental stuttering (Alm, 2004, 2021; Cai, Tourville, et al., 2014; Chang & Guenther, 2020), affecting the timing signaling of the PUT and reducing the automation of speech motor sequencing. Thus, if this motor impairment were to be the core difference in the speech motor network of CWS, then within-network connectivity involving the SMA, left vPMC, and left PUT may be reduced among these participants, or most significantly among CWSp. Furthermore, reduced functional connectivity between the left PUT and left SMA within the motor loop may also result in increased dependency on cortical-level connectivity between the left preSMA and left SMA for the initiation of speech motor programs and would manifest as significant increases in between-network connectivity crossing over between the planning and motor circuits in CWS.

We also hypothesized that CWS would show reduced centrality of nodes within the planning and motor loops, compared to those of CNS. Based on previous research findings (Cai, Tourville, et al., 2014; Chang et al., 2011; Chang & Zhu, 2013; Chow et al., 2023; Rowe et al., 2024), we anticipated reduced node centrality among CWS in the left pIFS and left caudate nucleus of the planning loop as well as in the left vPMC and left PUT of the motor loop. Further in line with previous evidence for increased recruitment of right hemispheric regions (De Nil et al., 2001; Fox et al., 1996; Neef et al., 2018) and reduced left-hemispheric connectivity (Chang et al., 2011; Chow & Chang, 2017; Watkins et al., 2008), we hypothesized that reduced node centrality for motor regions in the left hemisphere, including the left ventral primary motor cortex (M1), would also see increased node centrality for ROIs within the right hemisphere of CWS, specifically involving the right vPMC and right ventral primary motor (M1r) cortices. These right motor areas form core regions of the DIVA feedback control system, on which persons who stutter are thought to rely more heavily during speech motor production (Civier et al., 2010; Max et al., 2004).

## MATERIALS AND METHODS

### Participants

The child participants ranged from 3 to 10 years of age and were monolingual English speakers who performed within normal limits on standardized measures of language and cognitive performance. All participants took part in a larger neuroimaging study of developmental stuttering, where rsfMRI data were collected along with diffusion tensor imaging and structural MRI data. More information on study assessments and procedures can be found in previous publications (Chang et al., 2015; Chow et al., 2023). All study procedures were approved by the Institutional Review Board of Michigan State University. Informed consent to participate was obtained from all parents in written form and from all children in verbal (nonreaders or 3- to 5-year olds) or written form (readers, usually aged 6 years and older) in accordance with the Declaration of Helsinki.

Participants’ rsfMRI data were eligible for inclusion in the current study if they had at least 3 minutes of high-quality data, following motion censoring at frame displacement >0.5 mm and with a usable T1 image (*n* = 147). Seventy-three CWS and 74 age- and gender-matched CNS between 3.17 and 10.80 years of age were included in this study, following exclusion of 16 CWS and 10 CNS with less than 3 minutes of rsfMRI data, along with two further CWS and three CNS due to excessive motion. All participants took part in a larger longitudinal study. Stuttering was evaluated on an annual basis during their participation through the sampling of spontaneous speech, storytelling, and conversation, as well as reading for participants who were 6 years of age and older and were able to read, with all samples being video recorded. Stuttering behavior and severity were evaluated in accordance with the Stuttering Severity Instrument (SSI-4; Riley, 2009). Initially, stuttering status was diagnosed on the participant’s first visit and involved their composite SSI-4 score (≥10), their percentage of stuttering-like dysfluencies (SLDs) in these speech samples (SLD > 3%), as well as the expressed concern of the parent and confirmation by a speech-language pathologist. When neither the parent nor speech-language pathologist asserted stuttering and no family history of stuttering was reported, the child was considered as a CNS. Following the same criteria as previous studies (Yairi & Ambrose, 1999; Yairi et al., 1996), on subsequent visits, CWS were categorized as recovered when their SSI-4 score was below 10, percentage SLD was below 3, and the parent reported that the child’s stuttering status in the home setting and other environments had also changed to reflect these levels. In a few instances, some children exhibited lower than threshold-level stuttering frequency (e.g., SLD < 3%) but showed stuttered instances that were characteristic of stuttering (e.g., tense blocks). In these instances, even if SSI and percentage of SLD figures were low, the children were considered to be persistent if confirmed by parent and clinician reports. Alternatively, in a few instances some recovered children as well as control participants showed SLD exceeding 3%, with stuttered instances mostly comprising easy word repetitions. In these cases, the children were categorized as recovered if confirmed by parent and clinician reports. Thus, recovery was categorized on year 2, 3, or 4 of the child’s study participation. If persistence or recovery status remained unclear on the CWS’s final visit, the child’s speech status was revisited via parent interview by phone 1 to 2 years following the participant’s final visit. No children were recruited who had previously recovered. Any CWS who were categorized as recovered were confirmed to be recovered during the course of study participation or at follow-up interview with their parent.

The participant groups differed significantly on measures of IQ (Wechsler Preschool and Primary Scale of Intelligence [Wechsler, 2012] or Wechsler Abbreviated Scale of Intelligence [Wechsler, 1999]), with CWS scoring lower on average than CNS (though well within the normal range), as well as producing more SLDs. The groups did not differ on the measure of socioeconomic status (SES), which was based on parent educational attainment, usually that of the participant’s mother, on a seven-point scale derived from the Hollingshead Four Factor Index of Socioeconomic Status (Hollingshead, 1975). The demographics and behavioral scores of participants included in our final analyses are shown in Table 1. Exploratory analyses were also conducted between persistent and recovered CWS, with demographic information of these two groups (CWSp and CWSr) available in Table S1 in the Supplementary Materials, available at https://doi.org/10.1162/NOL.a.26. With the analysis of rsfMRI involving the first usable scan obtained from each participant, these scans were conducted in either year 1 (56 CWS, 64 CNS), year 2 (12 CWS, 7 CNS), or year 3 (5 CWS, 3 CNS) of study participation. As stuttering status was determined at enrollment, persistence and recovery determination for CWS’s analyzed rsfMRI data was made in a retrospective manner; while at the time of scanning, all CWS included in the study were considered as still exhibiting stuttering.

**Table 1.** Participant demographics and behavioral performance among CWS and CNS

	CNS	CWS	Group difference
# Participants (F:M)	39:35	34:39	
Age (yr)	5.96 ± 1.75	6.02 ± 1.93	*t*(145) = −0.30, *p* = 0.77
SES	6.23 ± 0.82	6.16 ± 0.80	*t*(145) = 0.49, *p* = 0.63
Composite IQ	112 ± 14.3	106 ± 13.8	*t*(145) = 2.57, *p* = 0.011
SLDs	1.02 ± 0.75	4.60 ± 2.94	*t*(81.28) = −10.08, *p* < 0.001
SSI-4		17.7 ± 6.30	

*Note*. Demographic and behavioral information were typically obtained at the first longitudinal study visit or corresponding to when the first high-quality fMRI scan was acquired for that participant. Group differences between children who do (CWS) and do not stutter (CNS) were calculated by independent sample *t* tests and Welch tests for samples of unequal variances. Socioeconomic status (SES) was measured by parent’s (mother’s) educational attainment, on a 7-point scale, and derived from the Hollingshead Four Factor Index of Socioeconomic Status (Hollingshead, 1975). Composite intelligence quotients (IQ) scores were based on performance on the Wechsler Preschool and Primary Scale of Intelligence (WPPSI; Wechsler, 2012) or the Wechsler Abbreviated Scale of Intelligence (WASI; Wechsler, 1999). Stuttering-like disfluencies (SLDs) were calculated by percentage of disfluent syllables during narrative and monologue speech samples of the “Frog, Where Are You?” picture book (Mayer, 1969). The composite stuttering severity rating was based on the Stuttering Severity Instrument–Fourth Edition (SSI-4; Riley, 2009) and calculated from the frequency, duration, and physical concomitants of stuttering moments during speech sampling.

### Neuroimaging Measures

#### MRI acquisition

Preceding all MRI scanning sessions, participants were trained during a separate visit with a mock scanner to familiarize and desensitize them to the sights and sounds of the scanner and to practice being still inside the scanner bore. During the rsfMRI scan, children lay supine with their eyes open and were instructed to remain as still as possible. An experimenter sat with the participant throughout the scanning session, to reassure the child and assist them in remaining calm, without moving. MRI scans were acquired on a GE 3T Signa HDx scanner (GE Healthcare) with an eight-channel head coil. One hundred eighty T1-weighted 1-mm^3^ isotropic volumetric inversion recovery fast spoiled gradient-recalled images (3D IRFSPGR) were obtained over the whole brain with cerebrospinal fluid suppressed in each scanning session (7 min scan time). For each scan, the following parameters were applied: time of echo (TE) = 3.8 ms, time of repetition of acquisition (TR) = 8.6 ms, time of inversion = 831 ms, repetition time of inversion = 2,332 ms, flip angle = 8°, field of view (FOV) = 25.6 cm × 25.6 cm, matrix size = 256 × 256, slice thickness = 1 mm, and receiver bandwidth = 20.8 kHz. For the rsfMRI acquisition, 36 contiguous 3 mm axial slices were collected with a gradient-echo echo-planar imaging sequence of 7 min in an interleaved order with TE = 27.7 ms, TR = 2.5 s, flip angle = 80°, FOV = 22 cm, matrix size = 64 × 64. From 164 time points collected, the first four data points were discarded.

#### Imaging data analysis

Data were processed using standard methods in Statistical Parametric Mapping (SPM12; Ashburner et al., 2021). Slice time was corrected using sinc-interpolation, and all scans were realigned to the 10th volume acquired during each scan. Time series of functional volumes were then co-registered with a high-resolution T1 image, spatially normalized to the MNI152 brain using the CAT12 toolbox, and then spatially smoothed with a 6 mm isotropic Gaussian kernel. ICA-AROMA (Pruim et al., 2015) was applied to the smoothed data for motion denoising. Resting-state processing steps were then applied, including linear detrending, CompCor (Behzadi et al., 2007), band-pass filtering from 0.1–0.01 Hz, and motion scrubbing of frames that exceed a framewise displacement of 0.5 mm. The same preprocessing pipeline was applied to all participants, including the youngest children. Although the pipeline was not explicitly tailored to account for age-related differences in brain morphology or motion, it incorporated standard motion correction and denoising steps (e.g., ICA-AROMA and motion scrubbing), which help reduce motion-related artifacts common in pediatric data.

Preprocessed time-series data (164 functional volumes) from each participant were then extracted from 12 regions of interest (ROIs). These were selected based on key regions in the GODIVA planning and motor loops (Bohland et al., 2010; Tourville, 2008), as well as the DIVA feedback and feedforward control systems (Guenther, 2016; Tourville & Guenther, 2011). The MNI coordinates for each ROI were first derived from research on this framework and images extracted through Neurosynth (https://www.neurosynth.org/)—an online tool that provides an automated meta-analytic synthesis of fMRI data from published research reports (Yarkoni et al., 2011). These were then preregistered online on the Open Science Framework (https://osf.io/c4t27). A sphere with a 5 mm radius around each set of Montreal Neurological Institute (MNI) coordinates was then used to create the CS-GIMME nodes for the 12 ROIs used in the current analyses, which are listed in Table 2.

**Table 2.** Cortical and subcortical regions of interest within MNI space and their functions in the DIVA and GODIVA networks

Regions of interest	MNI coordinates			Model function
GODIVA planning loop:				
left pre-supplementary motor area	−8	19	42	Sequential structure buffer
left posterior inferior frontal sulcus	−38	24	15	Phonological content buffer
left caudate nucleus	−12	−2	14	Receives input from planning loop
left ventral anterior thalamic nucleus	−8	−3	6	Receives output from planning loop
GODIVA motor loop:				
left supplementary motor area	0	0	68	Initiation map
left ventral premotor cortex	−56	10	2	Speech sound map
left putamen	−26	−2	4	Receives input from motor loop
left ventral lateral thalamic nucleus	−10	−14	8	Receives output from motor loop
DIVA feedforward control system:				
left ventral primary motor cortex	−53	0	42	Larynx articulator map
right superior lateral cerebellum lobule VI	12	−65	−15	Speech sound map to articulator map
DIVA feedback control system:				
right ventral premotor cortex	60	14	34	Feedback control map
right ventral primary motor cortex	53	4	42	Larynx articulator map

*Note*. Based on Guenther (2016) and Tourville (2008).

#### GIMME

In the current study, rsfMRI data were analyzed using the confirmatory subgrouping extension of GIMME, namely, CS-GIMME (Henry et al., 2019; Lane et al., 2019). GIMME iteratively fits person-specific unified structural equation models in a data-driven manner, estimating both lagged and contemporaneous directed connections between nodes. Variables are analyzed independently of their input order and can also be fit via backward selection or in a confirmatory way, as applied here (Beltz & Gates, 2017; Henry et al., 2019). We sought to estimate subgroup-specific connections within the speech motor network for a priori defined groups (CWS and CNS), by identifying connections between nodes that are present for the majority of individuals within the CWS and CNS groups. We also tested for hypothesized impairments in the speech network of CWS, reflecting individual-specific heterogeneity in connectivity. Using each participant’s network estimated by CS-GIMME, we determined within-network density, as a measure of functional efficiency, and node centrality, as the contribution of each ROI to network function.

**Figure 2.**
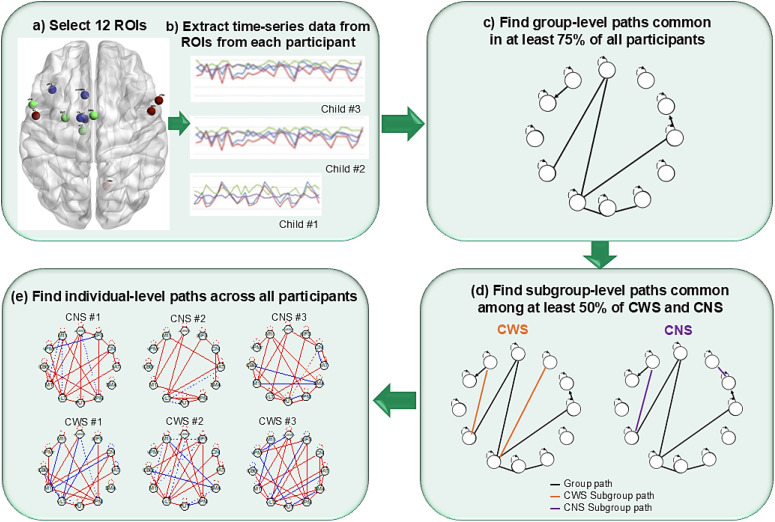
The analytic pipeline for confirmatory subgrouping group iterative multiple model estimation (CS-GIMME), adapted from Gates et al. (2017) . In step (A), 12 regions of interest (ROIs) were selected from the planning and motor circuits of the GODIVA model (Bohland et al., 2010), as well as the speech motor feedforward and feedback control systems defined by the DIVA model (Tourville & Guenther, 2011), with (B) resting-state fMRI time-series data (164 functional volumes) then extracted from each of the ROIs for each participant. In steps (C) through (E), the CS-GIMME algorithm was used to (C) identify group-level connections between the ROIs that were present in at least 75% of all participants, (D) then identify subgroup-level connections between the ROIs that were present in at least 50% of CWS or CNS participants, respectively, and (E) identify individual-level connections unique to a participant, resulting in the final networks containing group-, subgroup-, and individual-level lagged and contemporaneous connections. CWS = children who stutter, CNS = children who do not stutter.

The GIMME analysis pipeline produces group-level and individual-level connections within the network of each participant, involving the magnitude and direction of contemporaneous or lagged relations between nodes/ROIs (Beltz & Gates, 2017). In our application of CS-GIMME, the directionality of both contemporaneous and lagged connections was considered for all ROIs within the motor and planning loops. As described in Figure 2, CS-GIMME begins with a null model, containing only autoregressive connections for each node. Using Lagrange multiplier tests, the model then iteratively adds group-level (i.e., full sample), next subgroup-level (i.e., CWS vs. CNS), and finally individual-level connections to each participant’s network until their network model fits their functional data well, according to standard fit indices (see Gates & Molenaar, 2012). When applying CS-GIMME (Version 0.7.13) in R (Version 4.1.1), predefined classifications of individuals were considered during the model search procedure, using a 75% threshold for group-level edges and 50% for subgroup-level edges (Gates & Molenaar, 2012; Henry et al., 2019). Thus, as part of the confirmatory subgroup-level analysis, the threshold represents the minimum proportion of participants for whom a given network connection must significantly increase model fit in order for that connection to be estimated in everyone’s network. Significance is determined by modification indices (i.e., Lagrange multiplier tests with multiple comparison corrections), starting with group-level edges and then edges that improve model fit for individuals within a given subgroup (Henry et al., 2019).

#### Statistical analysis

Student *t* tests were initially conducted to compare group differences between CWS and CNS in network density and node centrality. Linear regression models were then used to statistically evaluate group differences in network density within the planning (including preSMA, pIFS, caudate, and ventral anterior thalamus) and motor loops (including SMA, vPMC, PUT, ventral lateral thalamus [vLT]), as well as differences in node centrality, with age, sex, SES, and IQ included as covariates. Post hoc analyses were conducted to examine significant group differences and their relationship to stuttering persistence by further categorizing CWS as either persistent (CWSp) or recovered (CWSr), as well as to identify possible effects of sex. In addition, linear regression models were employed to examine the relationship between stuttering severity and network density as well as node centrality, as indicated by group differences. Multiple comparisons were corrected using the false discovery rate (FDR) method implemented in R (stats Version 3.6.2; Benjamini & Hochberg, 1995). Comparisons of network density between the groups were hypothesis driven and were not corrected for multiple comparisons.

## RESULTS

**Figure 3.**
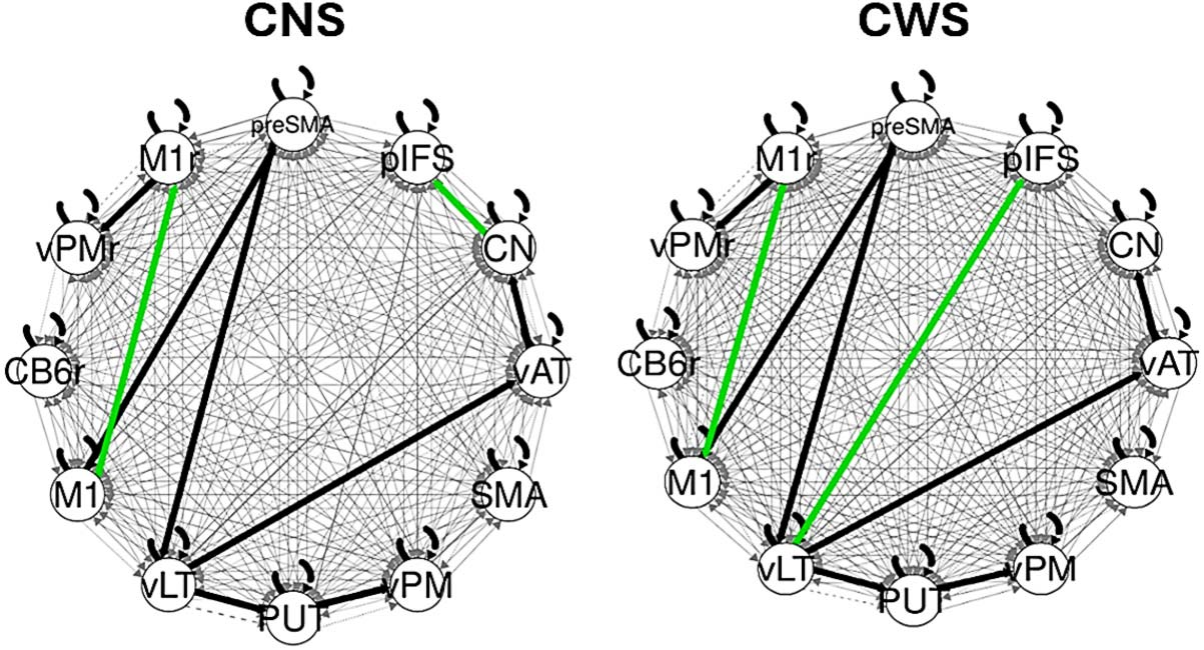
Summary connectivity maps derived from CS-GIMME for children who do not stutter (CNS) and children who stutter (CWS). The group-level connections common across the full participant sample are shown in thick black lines, while subgroup-specific increased connections are shown in green. Connections occurring at the individual-level are shown in grey lines, with line thickness reflecting the number of individual participants for whom a connection was estimated. Left: CNS showed a distinct connection between the left pIFS and CN, structures that are both within the planning loop. Right: CWS showed distinct connections between the planning loop node in the left pIFS and the motor loop node of the left vLT. Both groups contained primary motor cortex connections. CB6r = right superior lateral cerebellum lobule VI, CN = left caudate nucleus, M1 = left primary motor cortex, M1r = right primary motor cortex, pIFS = left posterior inferior frontal sulcus, preSMA = left pre-supplementary motor area, PUT = left putamen, vAT = left ventral anterior thalamic nucleus, vLT = left ventral lateral thalamic nucleus, vPM = left ventral premotor cortex, vPMr = right ventral premotor cortex, SMA = left supplementary motor area.

CS-GIMME detected group-level connections (present in both groups) among several ROIs of the speech motor network in the full sample, as illustrated by black weighted lines in Figure 3. Specifically, connections were observed within the planning loop between the left caudate nucleus and left ventral anterior thalamus, as well as within the motor loop between the left PUT and left vLT, and between the PUT and left vPMC. Group-level connections were also found between planning and motor loop ROIs, which included connectivity between the left preSMA and left vLT, as well as between the left ventral anterior thalamus and left vLT. Additional group-level connections were identified within the DIVA feedback control system between the right vPMC and right primary motor cortex (M1r), and between the planning and DIVA feedforward control circuits, involving the left preSMA and left M1.

Specific to CNS, a subgroup-level connection was identified between the left pIFS and left caudate nucleus, while subgroup-level connections between the left pIFS and left vLT were found to be specific to CWS. In addition, subgroup-level connections that differentiated CWS and CNS involved interhemispheric M1 connectivity, with predominantly M1 to M1r (left to right) connections found among CNS and M1r to M1 (right to left) among CWS.

### Network Density

**Figure 4.**
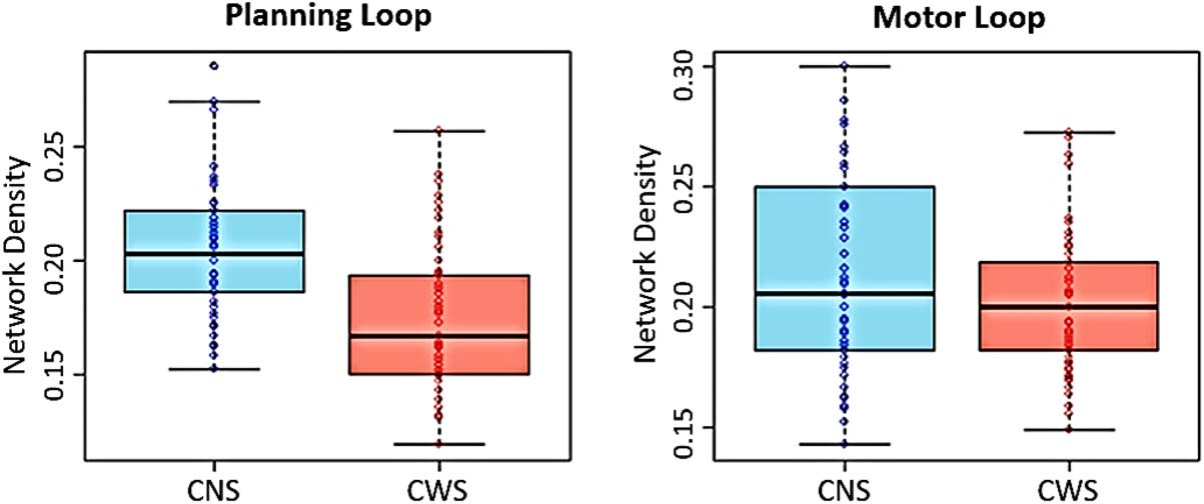
Group differences in within-network density between CNS (blue) and CWS (red) in the planning and motor loops.

CWS showed reduced within-network density in both the planning loop (*t* = 6.41, *p* < 0.0001) and the motor loop (*t* = 2.32, *p* = 0.022) when compared to CNS (Figure 4). However, when lagged connections were excluded, only the group difference for contemporaneous network connections within the planning loop remained significant (*t* = 6.65, *p* < 0.0001).

We further investigated whether the group difference in within-network density within the GODIVA planning loop could be mediated by age and sex (see Table S2 in the Supplementary Materials). A linear regression model that included age, sex, and their interactions with group, while controlling for SES and IQ, revealed a significant interaction between sex and group (*b* = 0.021, *SE* = 0.010, *t* = 2.18, *p* = 0.031), in addition to the main effect of group (*b* = −0.062, *SE* = 0.015, *t* = −4.01, *p* < 0.0001). Specifically, the group difference was more pronounced among girls (Group: *b* = 0.040, *SE* = 0.007, *t* = 5.89, *p* < 0.0001), compared to boys (Group: *b* = 0.019, *SE* = 0.007, *t* = 2.74, *p* = 0.007). These results are depicted in Figure S1 in the Supplementary Materials. No similar interaction was observed within the GODIVA motor loop.

**Figure 5.**
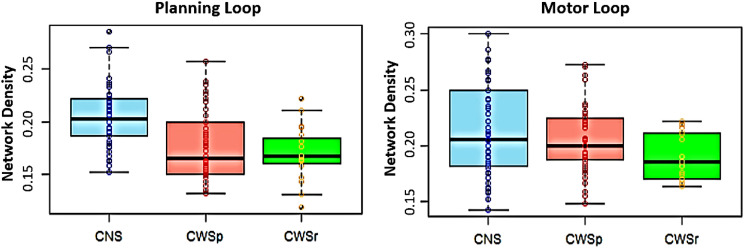
Group differences between CNS (blue), persistent CWS (CWSp; red) and recovered CWS (CWSr; green) in network density of the GODIVA planning loop and GODIVA motor loop were not significant.

Where significant subgroup level differences between CWS and CNS were identified, post hoc analyses were used to explore potential differences between CWSp and CWSr. Preliminary analyses showed no significant difference between these two groups in network density of the planning loop (*t* = 0.72, *p* = 0.48); however, a group effect approaching significance was found in the motor loop (*t* = 1.93, *p* = 0.06), as illustrated in Figure 5. No significant correlations were observed between stuttering severity and network density among CWS.

### Node Centrality

**Figure 6.**
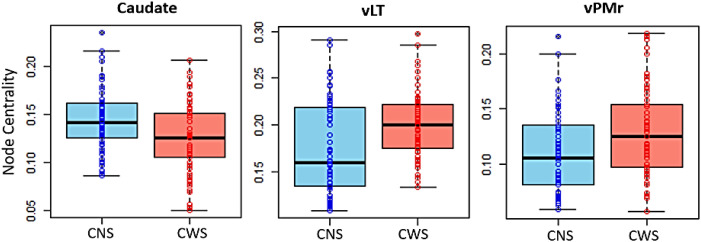
Group differences in node centrality between CNS (blue) and CWS (red) in the left caudate nucleus, left ventrolateral thalamus (vLT), and right ventral premotor region (vPMr).

Group differences in node centrality were found within the left caudate, left vLT and right ventral premotor regions. These remained significant following correction for multiple comparisons using FDR. Specifically, CWS exhibited reduced node centrality in the left caudate nucleus when compared to CNS (*t* = 3.348, *p*_adj_ = 0.004), but increased node centrality in the left vLT compared to CNS (*t* = −3.855, *p*_adj_ < 0.0001), as well as in right ventral premotor regions (*t* = −3.428, *p*_adj_ = 0.004), as seen in Figure 6. These findings were also significant when accounting for demographic and cognitive factors, including age, sex, SES, and IQ (see Table S3 in the Supplementary Materials). No significant group differences were found involving any of the other ROIs, or when investigating differences between CWSp and CWSr involving the three nodes between which the main group differences were found.

**Figure 7.**
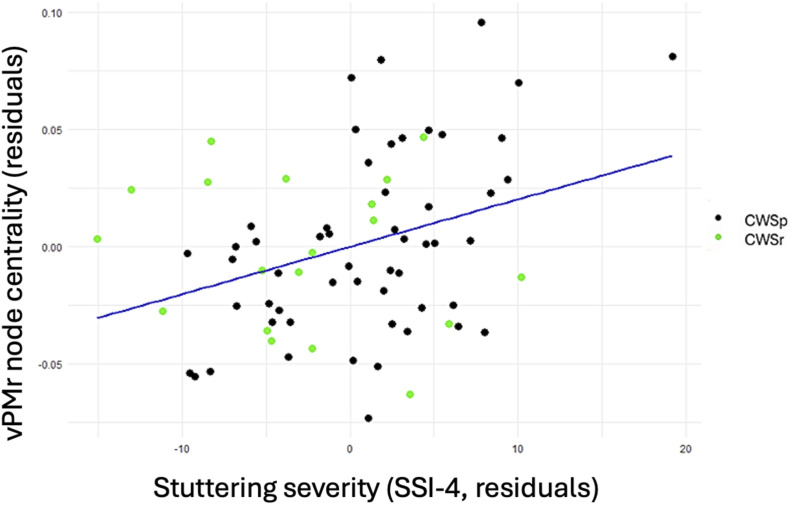
In CWS, stuttering severity, as measured by the Stuttering Severity Instrument–Fourth Edition (SSI-4; Riley, 2009), was positively associated with node centrality in the right ventral premotor cortex (vPMr). CWSr are represented by green dots and CWSp by black dots. Analyses were controlled for age, sex, socioeconomic status, and IQ.

Lastly, within the CWS group, a positive correlation between stuttering severity, as measured on the SSI-4, and node centrality was found in the right ventral premotor region (*b* = 0.002, *SE* = 0.0007, *t* = 2.931, *p* = 0.005; Figure 7). This correlation also remained significant without the inclusion of demographic and cognitive covariates, including age, sex, SES, and IQ (*b* = 0.002, *SE* = 0.0007, *t* = 3.053, *p* = 0.003). This suggests a potential association between the severity of stuttering and the functional centrality of brain regions responsible for processing corrective motor commands through feedback control mechanisms during speech. The difference in node centrality of the right vPMr between CWSp and CWSr was found to be statistically nonsignificant, however (*t* = 0.504 *p* = 0.617).

## DISCUSSION

In this study, we conducted a network-level analysis to compare resting-state functional connectivity within the speech motor networks of CWS and CNS. We hypothesized that network density within the planning and motor circuits of CWS would be significantly reduced as compared to CNS. When accounting for individual specificity in network connectivity, CWS showed significant alterations in both within- and between-network connectivity, compared to CNS. These differences implicated both the GODIVA planning and motor loops, as well as critical cortical and subcortical connections in the BGTC circuitry. Specifically, subgroup analyses revealed a significant reduction in network density within each of the planning and motor loops in CWS compared to CNS, supporting our hypothesis. This reduction was more pronounced in the planning loop and was most significant among girls who stutter.

### Reduced Optimization of Speech Planning and Motor Networks in CWS

Network density reflects the efficiency and strength of within-network communication, and its reduction in CWS in the present results suggests weakened connectivity critical to speech motor planning. It has previously been hypothesized that the core deficit in developmental stuttering is associated with deficiencies within the GODIVA planning loop, affecting the ability to initiate, sustain, and/or terminate motor programs within a speech sequence (Guenther, 2016). Early in development, projections from the left preSMA are essential for initiating phonemic segments within the intended syllabic structure in the left SMA initiation map, before the basal ganglia motor loop assumes control over these acquired motor programs (Meier & Guenther, 2023). Increased connectivity observed among CWS between the left pIFS, an area also supporting working memory, and the left vLT, may reflect a maladaptive optimization of neural networks (Beltz & Gates, 2017). In this case, forming inefficient functional connections that cross over between the planning and motor circuits could possibly indicate the need for more cognitive (prefrontal) involvement in generating speech motor output in CWS. Together, these findings suggest that developmental stuttering may be associated with reduced optimization of neural networks, particularly in regions that support sequencing of speech sounds, as well as having a continued reliance on working memory resources in the left pIFS. This reduced optimization may lead to ineffective automation of the speech sequencing process, which is achieved during early childhood, when control of well-learned speech motor sequences is assumed by the BGTC circuitry (Alm, 2004, 2021; Chang & Guenther, 2020; Guenther, 2016; Meier & Guenther, 2023).

Most recently, Rowe et al. (2024) applied an unsupervised clustering technique of principal component analysis to resting-state functional connectivity among AWS and ANS, and reported significant group distinctions in the GODIVA planning loop, as seen in the connections between the left thalamus and left pIFS, and in the GODIVA motor loop, between the left SMA and left vPMC. Significant increases in connectivity within the planning loop and reduced connectivity in the motor loop were proposed to support the subtyping of persistent developmental stuttering, with these affecting the planning and motor systems of persons who stutter differentially. Similarly, the density of within-network connectivity was reduced in the motor loop of CWS in the current study, while the planning loop showed the most significant reduction in network density among CWS, as compared to CNS. While Rowe and colleagues found increased connectivity between the left thalamus and left pIFS of the planning loop in a subgroup of AWS, we found heightened connectivity between the left vLT of the motor loop and left pIFS of the planning loop. One could infer that inefficient cross-network connectivity during childhood may result in compensatory mechanisms creating a heightened reliance on processing within the speech planning circuit into adulthood, while differences in within network connectivity of the motor loop show earlier indications of reduced functional optimization, as seen in our group of CWS. These findings may indicate within-group heterogeneity among CWS, as well as reflecting a critical reliance on the planning loop as part of early speech motor skill acquisition, to provide long-term efficiencies in speech production. While our application of GIMME involved the fitting of participant-specific unified structural equation models at the group, subgroup, and individual levels, the subgroups in the current investigation were identified a priori (as CNS and CWS). The identification of subgroups or subtypes within CWS, as was done in Rowe et al. (2024), was not possible by use of the current CS-GIMME approach; however, future investigations may look toward individual-level analyses through use of GIMME to help identify individual specific connectivity patterns and how they relate to aspects of stuttering relevant behavior.

The observed increased connectivity involving the right vPMC among our group of CWS supports the theory that developmental stuttering may involve an over-reliance on auditory feedback control, for which the right vPMC receives error signals from sensory regions (Civier et al., 2010; Tourville & Guenther, 2011). These error signals are transformed into corrective motor commands via projections to the left motor cortex (M1). However, our interpretation of these findings can only be speculative when no additional components from the DIVA feedback control system were included in the current investigation. Nevertheless, behavioral research among AWS supports the notion that stuttering is associated with differences in auditory integration processes during speech, whereby adults with persistent stuttering have shown reduced modulation of the auditory system during speech planning (Cai, Tourville, et al., 2014; Daliri & Max, 2015b), as well as reduced lateralization of activity within the auditory feedback system (Daliri & Max, 2015a). Recent evidence also suggests that feedback control may involve differential modulation across the hemispheres, with the right hemisphere supporting spectral feedback control in addition to left hemispheric temporal feedback control of auditory information (Floegel et al., 2020). Thus, further research into the contribution of right vPMC to spectral feedback control in persons who stutter is required in order to better understand its role in developmental stuttering.

In the present study, no significant group differences were found in node centrality of the left vPMC between CWS and CNS, while group level connections between left PUT and left vPMC were found across participants, and node centrality of the right vPMC did distinguish the groups. CWS showed an increased relative number of connections to the right vPMC, as compared to CNS, which was also found to correlate positively with stuttering severity. These findings indicate early recruitment of the right vPMC—a region that is central to feedback control and responsible for receiving error signals from the posterior auditory cortex and ventral somatosensory cortex for the online sensorimotor control of speech production. The association of increased node centrality of the feedback control map with increased severity of stuttering behavior in childhood stuttering suggests that CWS may become dependent on sensorimotor feedback control mechanisms during speech, while the decreased efficiency of node-to-node communication involving the left vPMC speech sound map may become more significant with stuttering persistence, as was found by Cai, Tourville, et al. (2014). An important caveat when comparing results from the study by Cai et al. and those from the current study is that the two studies not only differed in the age of the participants (adults and children, respectively), but the former focused on WM structural connectivity rather than functional connectivity that was investigated in the current study. Specifically, Cai et al. applied network-based statistics and graph theory to analyze the connectivity patterns obtained from tractography, whereas the current study used GIMME to analyze functional connectivity patterns occurring in rsfMRI data. Hence, the potential developmental differences in the left and right vPMC node centrality relevant to stuttering warrants further investigation, possibly through multimodal neuroimaging investigations spanning adults and children.

### Anomalous Cortico-Striatal Connectivity in CWS

We had also hypothesized that if developmental stuttering were to arise from disruptions to the planning loop, then connectivity involving the left preSMA, left pIFS, and left caudate would be significantly reduced in CWS, or among those who developed ineffective compensatory mechanisms and persisted to stutter. In addition, we hypothesized that this disruption of basal ganglia involvement could result in increased dependency on corticocortical connectivity between the left preSMA and left SMA for the initiation of speech motor programs, reflected in increased cross-circuit connectivity among CWS. We found that CNS showed distinct group-level connectivity between the left pIFS and left caudate, within the planning loop—functional connectivity that was not significant for CWS. We also found cross-circuit connectivity between the motor and planning loops of CWS; however, this was not found between the left preSMA and left SMA. Cross-cortical connectivity was found between the primary motor areas (M1), which indicated differential directionality between the groups.

Within the GODIVA framework of speech sequencing, the connection between the phonological sequence representation in the left pIFS and the left caudate (evident in CNS and summarized in Figure 3) constitutes a functional connection within the planning loop. This connection ensures that input received from the left preSMA via the left caudate activates the selected phonological output in the left pIFS, while suppressing other competing planning representations during speech production (Bohland et al., 2010). Connectivity between these model components was specific to CNS, while the cross-circuit connectivity between the left pIFS and left vLT was specific to CWS. The left vLT receives output projections from the left vPMC, and heavily projects to left primary motor cortex. When stimulated, the left vLT can affect speech rate, loudness, compulsion or even lead to speech arrest (as reviewed in Guenther, 2016). Its connection to the phonological content buffer in the left pIFS may further suggest that impaired cortico-striatal connectivity in the planning loop causes CWS to rely more heavily on prefrontal cortex (in particular, left pIFS), rather than PMC and/or basal ganglia, to initiate speech motor output.

Impaired cortico-striatal function in CWS aligns with previous research by Foundas et al. (2013) and Miller et al. (2023), both of whom reported reduced GMV in the right caudate. Foundas et al. (2013) also found atypical leftward asymmetry in caudate volume among their sample of boys who stutter, as well as demonstrating less typical hand laterality. Girls who stutter were not included in this earlier research. The authors suggested that these anomalies were reflective of associated vulnerabilities in motor planning and preparation, as well as inefficiencies in action-perception coupling due to deficiencies in the cortico-striato-thalamo-cortical feedforward networks (Foundas et al., 2013). Similarly, research in AWS has also reported reduced GMV in the left caudate nucleus when compared to ANS, further highlighting the implications of striatal dysfunction in the basal ganglia circuitry among individuals with developmental stuttering (Sowman et al., 2017).

### Increased Connectivity Within the Feedback Control Systems of CWS

In addition to these group-level differences, CWS exhibited increased centrality involving the vLT and right vPMC, when compared to CNS. The increased centrality of the left vLT found in CWS also aligns with the increased connectivity of that region to the left pIFS in CWS, which was not observed in CNS, shown in the connectivity map (see Figure 3). Within the GODIVA framework, the left vLT (see Figure 1), is part of the motor loop and is one of the key structures that supports transmission of initiation and termination commands for motor programs to the left SMA. The vLT is also affected by basal ganglia functioning, from which it receives substantial input. In developmental stuttering, failures of the basal ganglia in monitoring the current motor context (as represented in left vPMC, left SMA, and left vM1), may result in difficulties terminating the current phoneme, thereby resulting in a prolongation, or initiating the next phoneme and resulting in a block. A momentary drop of the signal from the basal ganglia via vLT to the left SMA may result in a premature termination and restart of the upcoming phoneme, manifesting as a repetition during speech production (Chang & Guenther, 2020; Guenther, 2016). It is of interest that this motor loop structure, as indicated in the GODIVA model, showed atypically greater connectivity with a planning loop structure (left pIFS) in CWS, which was not observed in CNS. This may indicate inefficiencies in the functioning of the motor loop in CWS.

The increased centrality of the left vLT in CWS compared to CNS can furthermore be interpreted, along with the observed results of increased centrality of the right vPMC, as involving increased dependency on feedback control mechanisms. Within the DIVA framework, the right vPMC projects indirectly via the pons, cerebellum, and left vLT to the articulator map in the left ventral motor cortex. Notably, the increased number of connections of the right vPMC was positively correlated with stuttering severity among CWS in this study, while the centrality of other nodes was not significantly correlated with stuttering. Within the DIVA framework, the right vPMC contains a lateralized feedback control map which receives error signals when incoming sensory feedback does not fall within the expected target region (Tourville & Guenther, 2011). These error signals are transformed into corrective motor commands via projections to the left M1. Previous research has also found increased rightward activation in right motor areas, including M1, SMA, and cingulate motor area, in persons who stutter (Brown et al., 2005; De Nil et al., 2008; Etchell et al., 2018). The increased connectivity observed in the right vPMC among our group of CWS supports the theory that developmental stuttering involves over-reliance on auditory feedback control, likely because poor feedforward commands result in sensory errors that invoke the feedback control system (Civier et al., 2010; Max et al., 2004).

This view is also consistent with previous findings of impairments in left hemisphere planning and premotor structures thought to be responsible for feedforward control, including the left IFG, specifically BA44, and left premotor regions in adults with persistent stuttering, while connectivity between homologous regions in the right hemisphere was increased compared to ANS (Chang et al., 2011; Watkins et al., 2008). However, Miller et al. (2023) recently investigated structural morphometry in children and adults with persistent stuttering and found cortical thickness to be reduced in speech planning regions, including the left midPMC, left vPMC, and left anterior central operculum, specifically among CWS. As previously mentioned, earlier research with AWS also showed reduced node betweenness centrality in the left vPMC among adults with persistent stuttering, when compared to the heightened centrality of this node among ANS (Cai, Tourville, et al., 2014). Added to this, Cler and colleagues (2021) found indications of greater iron concentration (as indicated by higher gray matter R2*) among AWS compared to ANS in the left frontal opercular cortex, left IFG (pars opercularis), IFS, the left PUT, and the left ventral precentral gyrus. These findings of greater iron concentration point toward implications for dopamine functioning within the BGTC circuitry and speech motor network of adults experiencing persistent developmental stuttering. White matter integrity, as measured by FA, underlying both left and right vPMC has also been found to be reduced in AWS (Watkins et al., 2008). Among adults, these findings implicate the left vPMC and the contribution of the speech sound map in the GODIVA motor loop, where input is received from the left SMA to initiate and program the speech motor sequences through its projections to the PUT, globus pallidus, and thalamus (Bohland et al., 2010; Guenther, 2016). Together, previous findings from network-based and tract-based spatial statistics, in addition to methods of FA averaging in AWS, highlight the connectivity deficiencies of these speech planning regions among persons who stutter. These may be associated with the underlying differences in functional connectivity of the speech planning circuitry seen in the left hemisphere of CWS in the current study, while also lending support to previous findings of over-reliance on feedback control mechanisms among persons who stutter in the right hemisphere.

### Differences in Connectivity to Primary Motor Regions Between CWS and CNS

Our investigation of node centrality using CS-GIMME revealed interhemispheric connectivity between the left and right primary motor regions that also suggested differential connectivity patterns between CWS and CNS. We had hypothesized that in addition to reduced centrality of nodes participating in the GODIVA planning and motor loops, CWS would show reduced centrality of nodes in motor regions of the left hemisphere, along with increased centrality of nodes within the right hemisphere.

Although the reliability and validity of directionality has yet to be verified, an increased rightward connectivity found among CWS corroborates previous research reports of increased recruitment of right hemispheric speech motor regions among persons who stutter (De Nil et al., 2001; Fox et al., 1996; Neef et al., 2018), while left-hemispheric connectivity was reportedly reduced (Chang et al., 2011; Chow & Chang, 2017; Watkins et al., 2008). AWS have also demonstrated a lack of left-hemisphere facilitation during fluent production of speech, with the extent of leftward recruitment also correlating negatively with their frequency of stuttering (Neef et al., 2015). Previous research in childhood stuttering found differences in cortical morphology that distinguished CWS from CNS, with CWSp having significantly decreased cortical thickness of the left ventral M1 than CNS or CWSr (Garnett et al., 2018). Increased rightward connectivity to the motor regions of the feedback control system may also reflect increased and early reliance of CWS on the processes of auditory and somatosensory feedback control during speech (Civier et al., 2010; Max et al., 2004). Anomalous neurodevelopment involving left M1 has been found as a neural hallmark of developmental stuttering (Chang et al., 2019) and is hypothesized to contribute to impairments in cortical processing during speech production (Chang & Guenther, 2020). We interpret the current findings with caution, however, until the reliability and validity of this directionality in CWS can be verified through further modeling and future replication.

### Strengths, Limitations, and Future Directions

In this study, we leveraged the advanced and reliable network-level analytical approach of CS-GIMME to evaluate the motor and planning circuits in the speech motor networks of a substantial cohort of 73 CWS, comparing their individual network connectivity to that of 74 age- and sex-matched CNS. By these means, we were able to account for individual specificity of functional connectivity and identify both within- and between-network group differences that implicate the BGTC circuitry, as well as corticocortical-level connectivity, which support the development and increasing efficiency of speech motor control processes. The use of GIMME to study young persons who stutter also contributes to the growing evidence of heterogeneity among this population, as individual-level connectivity is accounted for within model estimations. This also serves as an important first step for follow-up studies to implement a data-driven approach to subgrouping of neural networks, for the potential identification of subtypes of developmental stuttering as they emerge in early childhood.

One limitation of the current investigation involves the small number of participants in our group of CWS who had been verified as having recovered from stuttering (*n* = 16). No significant group differences in network density or node centrality were found between the CWS who recovered from stuttering and those who were reported as being persistent. Thus, we cannot offer additional information to the body of evidence that has previously elucidated the differential neurodevelopmental patterns underlying the potential neurophysiological mechanisms that contribute to stuttering persistence or recovery (Chow & Chang, 2017; Chow et al., 2023; Garnett et al., 2018). Caution must also be taken regarding our findings of differential directionality of the connections between the left and right hemisphere M1 nodes of CWS and CNS. While simulations using GIMME have been able to correctly identify the direction of contemporaneous connections for up to 90% of connections (Gates & Molenaar, 2012), accuracy has been shown to vary based on simulation features, including time series length and the proportion of lagged-to-contemporaneous connections. Therefore, the significance of differential directionality, such as that detected between the primary motor cortices for the participant groups in the current study, is interpreted with a high degree of caution. Further investigations that consider the directionality of individual-level functional connectivity by use of GIMME can help support the reliability and validity of these estimates.

An additional limitation of our research focus is the constrained number of ROIs that were selected as network nodes. While the number of estimated edges in this study was based on optimal estimates reported in previous applications of GIMME (Beltz & Gates, 2017) and prioritized in accordance with their significant contributions to the GODIVA and DIVA models (Bohland et al., 2010; Tourville & Guenther, 2011), the cross-network connectivity found within our group of CWS may reflect anomalous development of functional pathways that extend beyond the core ROIs of the speech motor network. Indeed, evidence of anomalous network connectivity within and across attentional networks has previously been reported among CWS, indicating that stuttering is a complex neurodevelopmental condition that has implications beyond speech motor production and its neural nodes (Chang et al., 2018). More expansive investigations can better support our growing understanding of the diversity and variability of the stuttering experience (Constantino et al., 2016; Tichenor & Yaruss, 2019).

Future research can address these limitations with closer investigation of additional regions participating in both feedback and feedforward control subsystems among children and adults who stutter. For example, the nodes that form the auditory feedback control subsystem, within the posterior auditory cortex and those that participate in somatosensory feedback control were not included among the ROIs in our analyses. Therefore, we may infer an increased reliance on feedback control systems in CWS overall, but to which extent this involves auditory or somatosensory feedback has yet to be distinguished. Among AWS, speech-related functional connectivity to the posterior auditory cortex was previously found to be reduced, while connectivity to the supramarginal gyrus within the somatosensory cortex was increased, when compared to ANS (Kell et al., 2018). These findings were specifically reported among males with persistent developmental stuttering (Kell et al., 2018); whereas functional connectivity to the auditory cortex has not been found to differ between larger groups of AWS and ANS of both sexes (Chang et al., 2011). Future research endeavors will benefit not only from the inclusion of regions in the extended speech network but also from the considered estimation of individual- as well as subgroup-level distinctions that can shed further light on the within-group heterogeneity of persons experiencing developmental stuttering across the life-span.

### Conclusions

We used the network-based approach of CS-GIMME to estimate person-specific resting-state functional connectivity within the speech motor networks of CWS and CNS. Our analysis revealed significant within- and between-network connectivity differences between the groups. CWS exhibited reduced functional connectivity within both the planning and motor loops that support speech motor sequencing. The current findings expand on recent reports of within-group distinctions among persons who stutter, whereby the planning and motor circuits can be differentially affected and may result in distinct disfluency profiles among individuals who stutter. Furthermore, we found that functional connectivity was most significantly reduced within the planning circuit among girls who stutter in this study, suggesting differential development of functional connectivity patterns between boys and girls who stutter. Overall, reduced optimization of functional connectivity within the left-lateralized cortico-basal ganglia circuit further suggests a breakdown in the automated and effortless feedforward control of speech production and continued reliance on cortical processing and cognitive resources in developmental stuttering. Further research that accounts for the individual specificity of neurophysiological and behavioral symptomology is warranted, along with investigations into the continued development of these circuits as stuttering persists into adulthood.

## ACKNOWLEDGMENTS

The authors wish to thank all the children and parents who participated in this study. We also thank Valeria Caruso for her help in setting up a preliminary GIMME analysis pipeline for an earlier study. We also thank the reviewers for their insightful comments and critiques that helped improve the manuscript.

## FUNDING INFORMATION

Soo-Eun Chang, Foundation for the National Institutes of Health (https://dx.doi.org/10.13039/100000009), Award ID: R01DC011277.

## AUTHOR CONTRIBUTIONS

**Fiona Höbler**: Conceptualization: Supporting; Formal analysis: Lead; Writing – original draft: Lead; Writing – review & editing: Lead. **Yanni Liu**: Conceptualization: Supporting; Formal analysis: Lead; Visualization: Lead; Writing – review & editing: Supporting. **Adriene M. Beltz**: Conceptualization: Supporting; Formal analysis: Supporting; Writing – review & editing: Supporting. **Hannah C. Becker**: Formal analysis: Supporting; Writing – review & editing: Supporting. **Mike Angstadt**: Formal analysis: Supporting; Writing – review & editing: Supporting. **Frank H. Guenther**: Conceptualization: Supporting; Writing – review & editing: Supporting. **Soo-Eun Chang**: Conceptualization: Lead; Data curation: Lead; Funding acquisition: Lead; Investigation: Supporting; Resources: Lead; Writing – review & editing: Supporting.

## CODE AND DATA AVAILABILITY

All analysis codes used in this study are openly available at https://github.com/GatesLab/gimme. The datasets presented in this study can be found in the Open Science Framework online repository (https://osf.io/uv263).

## Supplementary Material



## TECHNICAL TERMS

Confirmatory subgrouping group iterative multiple model estimation (CS-GIMME): An advanced data-driven method used in network neuroscience and time series modeling (especially in fMRI research) to identify individual-, group-, and subgroup-level connectivity patterns across subjects.
Stuttering-like dysfluencies (SLDs): Speech interruptions that are characteristic of stuttering and are distinct from typical, nonpathological pauses or hesitations in speech. These types of disfluencies are considered core features of stuttering and are commonly used to help diagnose stuttering, especially in children. SLDs include part-word repetitions, single-syllable word repetitions, prolongations, and blocks.

## References

[bib1] Alexander, G. E., DeLong, M. R., & Strick, P. L. (1986). Parallel organization of functionally segregated circuits linking basal ganglia and cortex. Annual Review of Neuroscience, 9, 357–381. 10.1146/annurev.ne.09.030186.002041, 3085570

[bib2] Alm, P. A. (2004). Stuttering and the basal ganglia circuits: A critical review of possible relations. Journal of Communication Disorders, 37(4), 325–369. 10.1016/j.jcomdis.2004.03.001, 15159193

[bib3] Alm, P. A. (2021). The dopamine system and automatization of movement sequences: A review with relevance for speech and stuttering. Frontiers in Human Neuroscience, 15, Article 661880. 10.3389/fnhum.2021.661880, 34924974 PMC8675130

[bib4] Ashburner, J., Barnes, G., Chen, C.-C., Daunizeau, J., Flandin, G., Friston, K., Gitelman, D., Glauche, V., Henson, R., Hutton, C., Jafarian, A., Kiebel, S., Kilner, J., Litvak, V., Mattout, J., Moran, R., Penny, W., Phillips, C., Razi, A., … Zeidman, P. (2021). SPM12 manual. Wellcome Centre for Human Neuroimaging. https://www.fil.ion.ucl.ac.uk/spm/doc/manual.pdf

[bib5] Baxter, S., Johnson, M., Blank, L., Cantrell, A., Brumfitt, S., Enderby, P., & Goyder, E. (2016). Non-pharmacological treatments for stuttering in children and adults: A systematic review and evaluation of clinical effectiveness, and exploration of barriers to successful outcomes. Health Technology Assessment, 20(2), 1–302, v–vi. 10.3310/hta20020, 26767317 PMC4781644

[bib6] Beal, D. S., Gracco, V. L., Brettschneider, J., Kroll, R. M., & De Nil, L. F. (2013). A voxel-based morphometry (VBM) analysis of regional grey and white matter volume abnormalities within the speech production network of children who stutter. Cortex, 49(8), 2151–2161. 10.1016/j.cortex.2012.08.013, 23140891 PMC3617061

[bib7] Beal, D. S., Gracco, V. L., Lafaille, S. J., & De Nil, L. F. (2007). Voxel-based morphometry of auditory and speech-related cortex in stutterers. NeuroReport, 18(12), 1257–1260. 10.1097/WNR.0b013e3282202c4d, 17632278

[bib8] Beal, D. S., Lerch, J. P., Cameron, B., Henderson, R., Gracco, V. L., & De Nil, L. F. (2015). The trajectory of gray matter development in Broca’s area is abnormal in people who stutter. Frontiers in Human Neuroscience, 9, Article 89. 10.3389/fnhum.2015.00089, 25784869 PMC4347452

[bib9] Becker, H. C., Beltz, A. M., Himle, J. A., Abelson, J. L., Block, S. R., Taylor, S. F., & Fitzgerald, K. D. (2024). Changes in brain network connections after exposure and response prevention therapy for obsessive-compulsive disorder in adolescents and adults. Biological Psychiatry: Cognitive Neuroscience and Neuroimaging, 9(1), 70–79. 10.1016/j.bpsc.2023.09.009, 37820789 PMC10842137

[bib10] Behzadi, Y., Restom, K., Liau, J., & Liu, T. T. (2007). A component based noise correction method (CompCor) for BOLD and perfusion based fMRI. NeuroImage, 37(1), 90–101. 10.1016/j.neuroimage.2007.04.042, 17560126 PMC2214855

[bib11] Beltz, A. M., & Gates, K. M. (2017). Network mapping with GIMME. Multivariate Behavioral Research, 52(6), 789–804. 10.1080/00273171.2017.1373014, 29161187 PMC6181449

[bib12] Belyk, M., Kraft, S. J., & Brown, S. (2015). Stuttering as a trait or state—An ALE meta-analysis of neuroimaging studies. European Journal of Neuroscience, 41(2), 275–284. 10.1111/ejn.12765, 25350867 PMC13140564

[bib13] Belyk, M., Kraft, S. J., & Brown, S. (2017). Stuttering as a trait or a state revisited: Motor system involvement in persistent developmental stuttering. European Journal of Neuroscience, 45(4), 622–624. 10.1111/ejn.13512, 28191730

[bib14] Benjamini, Y., & Hochberg, Y. (1995). Controlling the false discovery rate: A practical and powerful approach to multiple testing. Journal of the Royal Statistical Society: Series B (Methodological), 57(1), 289–300. 10.1111/j.2517-6161.1995.tb02031.x

[bib15] Bohland, J. W., Bullock, D., & Guenther, F. H. (2010). Neural representations and mechanisms for the performance of simple speech sequences. Journal of Cognitive Neuroscience, 22(7), 1504–1529. 10.1162/jocn.2009.21306, 19583476 PMC2937837

[bib16] Bohland, J. W., Tourville, J. A., & Guenther, F. H. (2019). Neural bases of speech production. In W. F. Katz & P. F. Assmann (Eds.), The Routledge handbook of phonetics (1st ed., pp. 122–160). Routledge. 10.4324/9780429056253-7

[bib17] Brown, S., Ingham, R. J., Ingham, J. C., Laird, A. R., & Fox, P. T. (2005). Stuttered and fluent speech production: An ALE meta-analysis of functional neuroimaging studies. Human Brain Mapping, 25(1), 105–117. 10.1002/hbm.20140, 15846815 PMC6871755

[bib18] Cai, S., Beal, D. S., Ghosh, S. S., Guenther, F. H., & Perkell, J. S. (2014). Impaired timing adjustments in response to time-varying auditory perturbation during connected speech production in persons who stutter. Brain and Language, 129, 24–29. 10.1016/j.bandl.2014.01.002, 24486601 PMC3947674

[bib19] Cai, S., Beal, D. S., Ghosh, S. S., Tiede, M. K., Guenther, F. H., & Perkell, J. S. (2012). Weak responses to auditory feedback perturbation during articulation in persons who stutter: Evidence for abnormal auditory-motor transformation. PLOS One, 7(7), Article e41830. 10.1371/journal.pone.0041830, 22911857 PMC3402433

[bib20] Cai, S., Tourville, J. A., Beal, D. S., Perkell, J. S., Guenther, F. H., & Ghosh, S. S. (2014). Diffusion imaging of cerebral white matter in persons who stutter: Evidence for network-level anomalies. Frontiers in Human Neuroscience, 8, Article 54. 10.3389/fnhum.2014.00054, 24611042 PMC3920071

[bib21] Chang, S.-E., Angstadt, M., Chow, H. M., Etchell, A. C., Garnett, E. O., Choo, A. L., Kessler, D., Welsh, R. C., & Sripada, C. (2018). Anomalous network architecture of the resting brain in children who stutter. Journal of Fluency Disorders, 55, 46–67. 10.1016/j.jfludis.2017.01.002, 28214015 PMC5526749

[bib22] Chang, S.-E., Erickson, K. I., Ambrose, N. G., Hasegawa-Johnson, M. A., & Ludlow, C. L. (2008). Brain anatomy differences in childhood stuttering. NeuroImage, 39(3), 1333–1344. 10.1016/j.neuroimage.2007.09.067, 18023366 PMC2731627

[bib23] Chang, S.-E., Garnett, E. O., Etchell, A., & Chow, H. M. (2019). Functional and neuroanatomical bases of developmental stuttering: Current insights. Neuroscientist, 25(6), 566–582. 10.1177/1073858418803594, 30264661 PMC6486457

[bib24] Chang, S.-E., & Guenther, F. H. (2020). Involvement of the cortico-basal ganglia-thalamocortical loop in developmental stuttering. Frontiers in Psychology, 10, Article 3088. 10.3389/fpsyg.2019.03088, 32047456 PMC6997432

[bib25] Chang, S.-E., Horwitz, B., Ostuni, J., Reynolds, R., & Ludlow, C. L. (2011). Evidence of left inferior frontal–premotor structural and functional connectivity deficits in adults who stutter. Cerebral Cortex, 21(11), 2507–2518. 10.1093/cercor/bhr028, 21471556 PMC3183422

[bib26] Chang, S.-E., & Zhu, D. C. (2013). Neural network connectivity differences in children who stutter. Brain, 136(12), 3709–3726. 10.1093/brain/awt275, 24131593 PMC3859219

[bib27] Chang, S.-E., Zhu, D. C., Choo, A. L., & Angstadt, M. (2015). White matter neuroanatomical differences in young children who stutter. Brain, 138(3), 694–711. 10.1093/brain/awu400, 25619509 PMC4339778

[bib28] Chow, H. M., & Chang, S.-E. (2017). White matter developmental trajectories associated with persistence and recovery of childhood stuttering. Human Brain Mapping, 38(7), 3345–3359. 10.1002/hbm.23590, 28390149 PMC5632574

[bib29] Chow, H. M., Garnett, E. O., Koenraads, S. P. C., & Chang, S.-E. (2023). Brain developmental trajectories associated with childhood stuttering persistence and recovery. Developmental Cognitive Neuroscience, 60, Article 101224. 10.1016/j.dcn.2023.101224, 36863188 PMC9986501

[bib30] Civier, O., Bullock, D., Max, L., & Guenther, F. H. (2013). Computational modeling of stuttering caused by impairments in a basal ganglia thalamo-cortical circuit involved in syllable selection and initiation. Brain and Language, 126(3), 263–278. 10.1016/j.bandl.2013.05.016, 23872286 PMC3775364

[bib31] Civier, O., Tasko, S. M., & Guenther, F. H. (2010). Overreliance on auditory feedback may lead to sound/syllable repetitions: Simulations of stuttering and fluency-inducing conditions with a neural model of speech production. Journal of Fluency Disorders, 35(3), 246–279. 10.1016/j.jfludis.2010.05.002, 20831971 PMC2939043

[bib32] Cler, G. J., Krishnan, S., Papp, D., Wiltshire, C. E. E., Chesters, J., & Watkins, K. E. (2021). Elevated iron concentration in putamen and cortical speech motor network in developmental stuttering. Brain, 144(10), 2979–2984. 10.1093/brain/awab283, 34750604 PMC8634076

[bib33] Constantino, C. D., Eichorn, N., Buder, E. H., Beck, J. G., & Manning, W. H. (2020). The speaker’s experience of stuttering: Measuring spontaneity. Journal of Speech, Language, and Hearing Research, 63(4), 983–1001. 10.1044/2019_JSLHR-19-00068, 32213101

[bib34] Constantino, C. D., Leslie, P., Quesal, R. W., & Yaruss, J. S. (2016). A preliminary investigation of daily variability of stuttering in adults. Journal of Communication Disorders, 60, 39–50. 10.1016/j.jcomdis.2016.02.001, 26945438

[bib35] Cykowski, M. D., Fox, P. T., Ingham, R. J., Ingham, J. C., & Robin, D. A. (2010). A study of the reproducibility and etiology of diffusion anisotropy differences in developmental stuttering: A potential role for impaired myelination. NeuroImage, 52(4), 1495–1504. 10.1016/j.neuroimage.2010.05.011, 20471482 PMC4135434

[bib36] Daliri, A., & Max, L. (2015a). Electrophysiological evidence for a general auditory prediction deficit in adults who stutter. Brain and Language, 150, 37–44. 10.1016/j.bandl.2015.08.008, 26335995 PMC4663101

[bib37] Daliri, A., & Max, L. (2015b). Modulation of auditory processing during speech movement planning is limited in adults who stutter. Brain and Language, 143, 59–68. 10.1016/j.bandl.2015.03.002, 25796060 PMC4380808

[bib38] Daliri, A., Wieland, E. A., Cai, S., Guenther, F. H., & Chang, S.-E. (2018). Auditory-motor adaptation is reduced in adults who stutter but not in children who stutter. Developmental Science, 21(2), Article e12521. 10.1111/desc.12521, 28256029 PMC5581739

[bib39] De Nil, L. F., Beal, D. S., Lafaille, S. J., Kroll, R. M., Crawley, A. P., & Gracco, V. L. (2008). The effects of simulated stuttering and prolonged speech on the neural activation patterns of stuttering and nonstuttering adults. Brain and Language, 107(2), 114–123. 10.1016/j.bandl.2008.07.003, 18822455

[bib40] De Nil, L. F., Kroll, R. M., & Houle, S. (2001). Functional neuroimaging of cerebellar activation during single word reading and verb generation in stuttering and nonstuttering adults. Neuroscience Letters, 302(2–3), 77–80. 10.1016/S0304-3940(01)01671-8, 11290391

[bib41] Donaher, J., & Richels, C. (2012). Traits of attention deficit/hyperactivity disorder in school-age children who stutter. Journal of Fluency Disorders, 37(4), 242–252. 10.1016/j.jfludis.2012.08.002, 23218208

[bib42] Etchell, A. C., Civier, O., Ballard, K. J., & Sowman, P. F. (2018). A systematic literature review of neuroimaging research on developmental stuttering between 1995 and 2016. Journal of Fluency Disorders, 55, 6–45. 10.1016/j.jfludis.2017.03.007, 28778745

[bib43] Floegel, M., Fuchs, S., & Kell, C. A. (2020). Differential contributions of the two cerebral hemispheres to temporal and spectral speech feedback control. Nature Communications, 11(1), Article 2839. 10.1038/s41467-020-16743-2, 32503986 PMC7275068

[bib44] Foundas, A. L., Cindass, R., Jr., Mock, J. R., & Corey, D. M. (2013). Atypical caudate anatomy in children who stutter. Perceptual and Motor Skills, 116(2), 528–543. 10.2466/15.10.PMS.116.2.528-543, 24032328

[bib45] Fox, P. T., Ingham, R. J., Ingham, J. C., Hirsch, T. B., Downs, J. H., Martin, C., Jerabek, P., Glass, T., & Lancaster, J. L. (1996). A PET study of the neural systems of stuttering. Nature, 382(6587), 158–161. 10.1038/382158a0, 8700204

[bib46] Garnett, E. O., Chow, H. M., Nieto-Castañón, A., Tourville, J. A., Guenther, F. H., & Chang, S.-E. (2018). Anomalous morphology in left hemisphere motor and premotor cortex of children who stutter. Brain, 141(9), 2670–2684. 10.1093/brain/awy199, 30084910 PMC6113637

[bib47] Gates, K. M., Lane, S. T., Varangis, E., Giovanello, K., & Guiskewicz, K. (2017). Unsupervised classification during time-series model building. Multivariate Behavioral Research, 52(2), 129–148. 10.1080/00273171.2016.1256187, 27925768 PMC8549846

[bib48] Gates, K. M., & Molenaar, P. C. M. (2012). Group search algorithm recovers effective connectivity maps for individuals in homogeneous and heterogeneous samples. NeuroImage, 63(1), 310–319. 10.1016/j.neuroimage.2012.06.026, 22732562

[bib49] Gates, K. M., Molenaar, P. C. M., Hillary, F. G., Ram, N., & Rovine, M. J. (2010). Automatic search for fMRI connectivity mapping: An alternative to Granger causality testing using formal equivalences among SEM path modeling, VAR, and unified SEM. NeuroImage, 50(3), 1118–1125. 10.1016/j.neuroimage.2009.12.117, 20060050

[bib50] Gates, K. M., Molenaar, P. C. M., Iyer, S. P., Nigg, J. T., & Fair, D. A. (2014). Organizing heterogeneous samples using community detection of GIMME-derived resting state functional networks. PLOS One, 9(3), Article e91322. 10.1371/journal.pone.0091322, 24642753 PMC3958357

[bib51] Gracco, V. L., Sares, A. G., & Koirala, N. (2022). Structural brain network topological alterations in stuttering adults. Brain Communications, 4(2), Article fcac058. 10.1093/braincomms/fcac058, 35368614 PMC8971894

[bib52] Guenther, F. H. (2016). Neural control of speech. MIT Press. 10.7551/mitpress/10471.001.0001

[bib53] Guenther, F. H., & Vladusich, T. (2012). A neural theory of speech acquisition and production. Journal of Neurolinguistics, 25(5), 408–422. 10.1016/j.jneuroling.2009.08.006, 22711978 PMC3375605

[bib54] Henry, T. R., Feczko, E., Cordova, M., Earl, E., Williams, S., Nigg, J. T., Fair, D. A., & Gates, K. M. (2019). Comparing directed functional connectivity between groups with confirmatory subgrouping GIMME. NeuroImage, 188, 642–653. 10.1016/j.neuroimage.2018.12.040, 30583065 PMC6901282

[bib55] Holland, S. K., Vannest, J., Mecoli, M., Jacola, L. M., Tillema, J.-M., Karunanayaka, P. R., Schmithorst, V. J., Yuan, W., Plante, E., & Byars, A. W. (2007). Functional MRI of language lateralization during development in children. International Journal of Audiology, 46(9), 533–551. 10.1080/14992020701448994, 17828669 PMC2763431

[bib56] Hollingshead, A. B. (1975). Four-factor index of social status [Unpublished manuscript]. Department of Sociology, Yale University.

[bib57] Jäncke, L., Hänggi, J., & Steinmetz, H. (2004). Morphological brain differences between adult stutterers and non-stutterers. BMC Neurology, 4(1), Article 23. 10.1186/1471-2377-4-23, 15588309 PMC539354

[bib58] Kell, C. A., Neumann, K., Behrens, M., von Gudenberg, A. W., & Giraud, A.-L. (2018). Speaking-related changes in cortical functional connectivity associated with assisted and spontaneous recovery from developmental stuttering. Journal of Fluency Disorders, 55, 135–144. 10.1016/j.jfludis.2017.02.001, 28216127

[bib59] Kell, C. A., Neumann, K., von Kriegstein, K., Posenenske, C., von Gudenberg, A. W., Euler, H., & Giraud, A.-L. (2009). How the brain repairs stuttering. Brain, 132(10), 2747–2760. 10.1093/brain/awp185, 19710179

[bib60] Lane, S. T., Gates, K. M., Pike, H. K., Beltz, A. M., & Wright, A. G. C. (2019). Uncovering general, shared, and unique temporal patterns in ambulatory assessment data. Psychological Methods, 24(1), 54–69. 10.1037/met0000192, 30124300 PMC6433550

[bib61] Lu, C., Chen, C., Peng, D., You, W., Zhang, X., Ding, G., Deng, X., Yan, Q., & Howell, P. (2012). Neural anomaly and reorganization in speakers who stutter: A short-term intervention study. Neurology, 79(7), 625–632. 10.1212/WNL.0b013e31826356d2, 22875083

[bib62] Lu, C., Peng, D., Chen, C., Ning, N., Ding, G., Li, K., Yang, Y., & Lin, C. (2010). Altered effective connectivity and anomalous anatomy in the basal ganglia-thalamocortical circuit of stuttering speakers. Cortex, 46(1), 49–67. 10.1016/j.cortex.2009.02.017, 19375076

[bib63] Ludlow, C. L. (2000). Stuttering: Dysfunction in a complex and dynamic system. Brain, 123(10), 1983–1984. 10.1093/brain/123.10.1983, 11004116

[bib64] Ludlow, C. L., & Loucks, T. (2003). Stuttering: A dynamic motor control disorder. Journal of Fluency Disorders, 28(4), 273–295. 10.1016/j.jfludis.2003.07.001, 14643066

[bib65] Masapollo, M., Segawa, J. A., Beal, D. S., Tourville, J. A., Nieto-Castañón, A., Heyne, M., Frankford, S. A., & Guenther, F. H. (2021). Behavioral and neural correlates of speech motor sequence learning in stuttering and neurotypical speakers: An fMRI investigation. Neurobiology of Language, 2(1), 106–137. 10.1162/nol_a_00027, 34296194 PMC8294667

[bib66] Matsuhashi, K., Itahashi, T., Aoki, R., & Hashimoto, R.-I. (2023). Meta-analysis of structural integrity of white matter and functional connectivity in developmental stuttering. Brain Research Bulletin, 205, Article 110827. 10.1016/j.brainresbull.2023.110827, 38013029

[bib67] Mattoni, M., Smith, D. V., & Olino, T. M. (2023). Characterizing heterogeneity in early adolescent reward networks and individualized associations with behavioral and clinical outcomes. Network Neuroscience, 7(2), 787–810. 10.1162/netn_a_00306, 37397889 PMC10312268

[bib68] Max, L., Guenther, F. H., Gracco, V. L., Ghosh, S. S., & Wallace, M. E. (2004). Unstable or insufficiently activated internal models and feedback-biased motor control as sources of dysfluency: A theoretical model of stuttering. Contemporary Issues in Communication Science and Disorders, 31(Spring), 105–122. 10.1044/cicsd_31_S_105

[bib69] Mayer, M. (1969). Frog, where are you? Dial Press.

[bib70] Meier, A. M., & Guenther, F. H. (2023). Neurocomputational modeling of speech motor development. Journal of Child Language, 50(6), 1318–1335. 10.1017/S0305000923000260, 37337871 PMC10615680

[bib71] Miller, H. E., Garnett, E. O., Heller Murray, E. S., Nieto-Castañón, A., Tourville, J. A., Chang, S.-E., & Guenther, F. H. (2023). A comparison of structural morphometry in children and adults with persistent developmental stuttering. Brain Communications, 5(6), Article fcad301. 10.1093/braincomms/fcad301, 38025273 PMC10653153

[bib72] Mink, J. W. (1996). The basal ganglia: Focused selection and inhibition of competing motor programs. Progress in Neurobiology, 50(4), 381–425. 10.1016/S0301-0082(96)00042-1, 9004351

[bib73] Molenaar, P. C. M. (2004). A manifesto on psychology as idiographic science: Bringing the person back into scientific psychology, this time forever. Measurement: Interdisciplinary Research and Perspectives, 2(4), 201–218. 10.1207/s15366359mea0204_1

[bib74] Neef, N. E., Anwander, A., Bütfering, C., Schmidt-Samoa, C., Friederici, A. D., Paulus, W., & Sommer, M. (2018). Structural connectivity of right frontal hyperactive areas scales with stuttering severity. Brain, 141(1), 191–204. 10.1093/brain/awx316, 29228195 PMC5837552

[bib75] Neef, N. E., Anwander, A., & Friederici, A. D. (2015). The neurobiological grounding of persistent stuttering: From structure to function. Current Neurology and Neuroscience Reports, 15(9), Article 63. 10.1007/s11910-015-0579-4, 26228377

[bib76] Ostry, D. J., Feltham, R. F., & Munhall, K. G. (1984). Characteristics of speech motor development in children. Developmental Psychology, 20(5), 859–871. 10.1037/0012-1649.20.5.859

[bib77] Price, R. B., Gates, K., Kraynak, T. E., Thase, M. E., & Siegle, G. J. (2017). Data-driven subgroups in depression derived from directed functional connectivity paths at rest. Neuropsychopharmacology, 42(13), 2623–2632. 10.1038/npp.2017.97, 28497802 PMC5686504

[bib78] Pruim, R. H. R., Mennes, M., van Rooij, D., Llera, A., Buitelaar, J. K., & Beckmann, C. F. (2015). ICA-AROMA: A robust ICA-based strategy for removing motion artifacts from fMRI data. NeuroImage, 112, 267–277. 10.1016/j.neuroimage.2015.02.064, 25770991

[bib79] Riley, G. D. (2009). SSI-4: Stuttering severity instrument—Fourth edition. PRO-ED.

[bib80] Rowe, H. P., Tourville, J. A., Nieto-Castanon, A., Garnett, E. O., Chow, H. M., Chang, S.-E., & Guenther, F. H. (2024). Evidence for planning and motor subtypes of stuttering based on resting state functional connectivity. Brain and Language, 253, Article 105417. 10.1016/j.bandl.2024.105417, 38703523 PMC11147703

[bib81] Ruland, S. H., Palomero-Gallagher, N., Hoffstaedter, F., Eickhoff, S. B., Mohlberg, H., & Amunts, K. (2022). The inferior frontal sulcus: Cortical segregation, molecular architecture and function. Cortex, 153, 235–256. 10.1016/j.cortex.2022.03.019, 35568575

[bib82] Segawa, J. A., Tourville, J. A., Beal, D. S., & Guenther, F. H. (2015). The neural correlates of speech motor sequence learning. Journal of Cognitive Neuroscience, 27(4), 819–831. 10.1162/jocn_a_00737, 25313656 PMC4344924

[bib83] SheikhBahaei, S., Millwater, M., & Maguire, G. A. (2023). Stuttering as a spectrum disorder: A hypothesis. Current Research in Neurobiology, 5, Article 100116. 10.1016/j.crneur.2023.100116, 38020803 PMC10663130

[bib84] Sitek, K. R., Cai, S., Beal, D. S., Perkell, J. S., Guenther, F. H., & Ghosh, S. S. (2016). Decreased cerebellar-orbitofrontal connectivity correlates with stuttering severity: Whole-brain functional and structural connectivity associations with persistent developmental stuttering. Frontiers in Human Neuroscience, 10, 190. 10.3389/fnhum.2016.00190, 27199712 PMC4855981

[bib85] Smith, A. (2006). Speech motor development: Integrating muscles, movements, and linguistic units. Journal of Communication Disorders, 39(5), 331–349. 10.1016/j.jcomdis.2006.06.017, 16934286

[bib86] Smith, A., & Weber, C. (2017). How stuttering develops: The multifactorial dynamic pathways theory. Journal of Speech, Language, and Hearing Research, 60(9), 2483–2505. 10.1044/2017_JSLHR-S-16-0343, 28837728 PMC5831617

[bib87] Sommer, M., Koch, M. A., Paulus, W., Weiller, C., & Büchel, C. (2002). Disconnection of speech-relevant brain areas in persistent developmental stuttering. Lancet, 360(9330), 380–383. 10.1016/S0140-6736(02)09610-1, 12241779

[bib88] Sowman, P. F., Ryan, M., Johnson, B. W., Savage, G., Crain, S., Harrison, E., Martin, E., & Burianová, H. (2017). Grey matter volume differences in the left caudate nucleus of people who stutter. Brain and Language, 164, 9–15. 10.1016/j.bandl.2016.08.009, 27693846

[bib89] Theys, C., Jaakkola, E., Melzer, T. R., De Nil, L. F., Guenther, F. H., Cohen, A. L., Fox, M. D., & Joutsa, J. (2024). Localization of stuttering based on causal brain lesions. Brain, 147(6), 2203–2213. 10.1093/brain/awae059, 38797521 PMC11146419

[bib90] Tichenor, S. E., Johnson, C. A., & Yaruss, J. S. (2021). A preliminary investigation of attention-deficit/hyperactivity disorder characteristics in adults who stutter. Journal of Speech, Language, and Hearing Research, 64(3), 839–853. 10.1044/2020_JSLHR-20-00237, 33647218

[bib91] Tichenor, S. E., & Yaruss, J. S. (2019). Stuttering as defined by adults who stutter. Journal of Speech, Language, and Hearing Research, 62(12), 4356–4369. 10.1044/2019_JSLHR-19-00137, 31830837

[bib92] Tourville, J. A. (2008). Neural mechanisms underlying auditory feedback control of speech [PhD dissertation]. Boston University. https://sites.bu.edu/guentherlab/files/2016/09/Tourville_Dissertation.pdf 10.1016/j.neuroimage.2007.09.054 PMC3658624 18035557

[bib93] Tourville, J. A., & Guenther, F. H. (2011). The DIVA model: A neural theory of speech acquisition and production. Language and Cognitive Processes, 26(7), 952–981. 10.1080/01690960903498424, 23667281 PMC3650855

[bib94] Watkins, K. (2011). Developmental disorders of speech and language: From genes to brain structure and function. In O. Braddick, J. Atkinson, & G. M. Innocenti (Eds.), Gene expression to neurobiology and behavior: Human brain development and developmental disorders (Vol. 189, pp. 225–238). Elsevier. 10.1016/B978-0-444-53884-0.00027-0, 21489392

[bib95] Watkins, K. E., Smith, S. M., Davis, S., & Howell, P. (2008). Structural and functional abnormalities of the motor system in developmental stuttering. Brain, 131(1), 50–59. 10.1093/brain/awm241, 17928317 PMC2492392

[bib96] Wechsler, D. (1999). WASI: Wechsler abbreviated scale of intelligence. Psychological Corporation. 10.1037/t15170-000

[bib97] Wechsler, D. (2012). WPPSI-IV: Wechsler preschool and primary scale of intelligence—Fourth edition. Pearson. 10.1037/spq0000038 24188289

[bib98] Yarkoni, T., Poldrack, R. A., Nichols, T. E., Van Essen, D. C., & Wager, T. D. (2011). Large-scale automated synthesis of human functional neuroimaging data. Nature Methods, 8(8), 665–670. 10.1038/nmeth.1635, 21706013 PMC3146590

[bib99] Yairi, E., & Ambrose, N. (2013). Epidemiology of stuttering: 21st century advances. Journal of Fluency Disorders, 38(2), 66–87. 10.1016/j.jfludis.2012.11.002, 23773662 PMC3687212

[bib100] Yairi, E., & Ambrose, N. G. (1999). Early childhood stuttering I: Persistency and recovery rates. Journal of Speech, Language, and Hearing Research, 42(5), 1097–1112. 10.1044/jslhr.4205.1097, 10515508

[bib101] Yairi, E., Ambrose, N. G., Paden, E. P., & Throneburg, R. N. (1996). Predictive factors of persistence and recovery: Pathways of childhood stuttering. Journal of Communication Disorders, 29(1), 51–77. 10.1016/0021-9924(95)00051-8, 8722529

