# Supplementary material for: The Speech Network in Childhood Stuttering: Differences in Functional Connectivity of the Planning and Motor Loops
Source: Neurobiol Lang (Camb). 2026 Jan 13;7:NOL.a.26. doi: 10.1162/NOL.a.26 (PMC12978680; doi:10.1162/NOL.a.26)
Supplement: Supplementary file 1 [file nol-07-26-s001.pdf]

Supplementary Materials

Table S1: Demographic data for children who do not stutter (CNS), children with persistent stuttering (CWSp) and children who recovered from stuttering (CWSr). These groups were compared in the exploratory analysis of subgroup-level differences.

	**CNS**	**CWSp**	**CWSr**
**# Participants (F:M)**	39:35	28:29	6:10
**Age (years)**	5.96 ± 1.75	6.19 ± 1.96	5.38 ± 1.74
**Socioeconomic Status (SES)**	6.23 ± 0.82	6.19 ± 0.81	6.06 ± 0.77
**Intelligence Quotients (IQ)**	112 ± 14.3	105 ± 13.5	109 ± 14.9
**Stuttering Like Dysfluencies (SLDs)**	1.02 ± 0.75	4.95 ± 3.04	3.32 ± 2.18
**SSI-4**		18.7 ± 5.99	14.3 ± 6.41

Demographic and behavioural information were typically obtained at the first longitudinal study visit or corresponding to when the first high-quality fMRI scan was acquired for that participant.

Table S2: Linear regression modelling of network density in the GODIVA *Planning Loop* between children who do (CWS) and do not stutter (CNS), including effects of age, sex, socioeconomic status (SES), and intelligence quotients (IQ), as well as interactions between age, group, and sex.

**Variable**	***b***	***SE***	***t***	***p***

**Model: GODIVA Planning Network Density.**				
Intercept	0.267	0.035	7.666	< .0001
Age	-0.000	0.001	-0.148	.882
Group	-0.062	0.015	-4.009	< .0001
Sex	-0.028	0.015	-1.849	.067
SES	-0.004	0.003	-1.470	.144
IQ	0.0003	0.000	1.765	.080
Age*Group	0.0004	0.001	0.525	.601
Age*Sex	-0.000	0.001	-0.055	.956
Group*Sex	0.021	0.010	2.176	.031
Age*Group*Sex	-0.000	0.000	-0.333	.740

Table S3: Linear regression modelling of node centrality in the left caudate nucleus (CN), left ventrolateral thalamus (vLT) and right ventral premotor regions (vPMr) between children who do (CWS) and do not stutter (CNS), when controlling for age, sex, socioeconomic status (SES), and intelligence quotients (IQ).

**Variable**	***b***	***SE***	***t***	***p***
**Model: CN Node Centrality.**				
Intercept	0.141	0.034	-4.211	< .001
Group	-0.018	0.006	-3.111	.002
Age	-0.000	0.000	-0.310	.757
Sex	-0.001	0.006	-0.138	.891
SES	0.002	0.004	0.530	.597

IQ	0.000	0.000	0.406	.685
**Model: vLT Node Centrality.**				
Intercept	0.194	0.042	4.654	< .001
Group	0.028	0.007	3.938	< .001
Age	0.000	0.000	0.105	.916
Sex	-0.010	0.007	-1.399	.164
SES	-0.009	0.004	-1.932	.055
IQ	0.000	0.000	0.721	.472
**Model: vPMr Node Centrality.**				
Intercept	0.078	0.037	2.169	.032
Group	0.020	0.006	3.222	.002
Age	0.000	0.000	1.322	.188
Sex	0.001	0.006	0.122	.903
SES	0.003	0.004	0.767	.445
IQ	-0.000	0.000	-0.455	.650

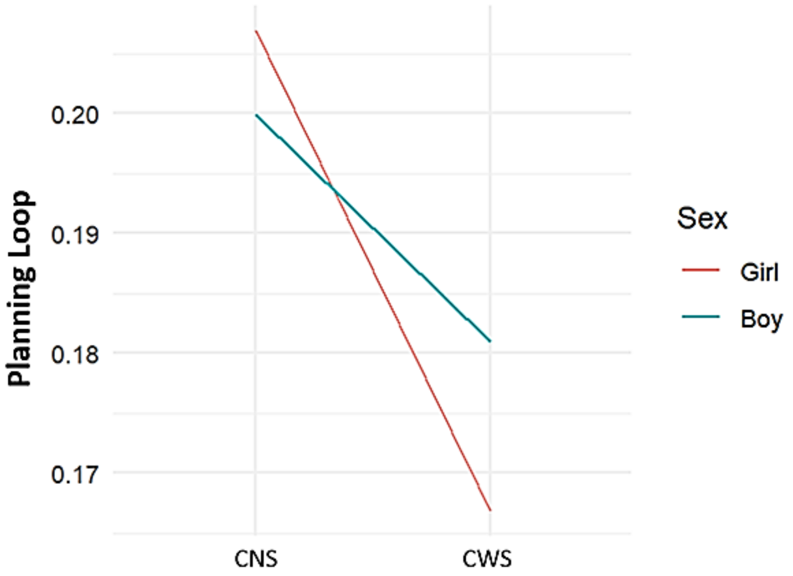

Figure S1. Between-group differences in network density of the GODIVA *Planning Loop* between boys (teal) and girls (coral) who do (CWS) and do not stutter (CNS). Girls who stutter showed the most significant reduction in network density of the GODIVA *Planning Loop* when compared to girls who do not stutter.
